# Seepage area of the cold seep exhibits strong homogeneous selection on prokaryotic community assembly and supports high depth variability of both archaeal and bacterial communities

**DOI:** 10.1128/spectrum.02722-24

**Published:** 2025-06-10

**Authors:** Xueling Xiong, Furun Li, Haokun Yang, Chunshan Li, Haiming Chen, Dan He, Qinglong L. Wu, Sijun Huang, Lijuan Ren

**Affiliations:** 1 Department of Ecology and Institute of Hydrobiology, Jinan University 47885, Guangzhou, China; 2 CAS Key Laboratory of Tropical Marine Bio-resources and Ecology, South China Sea Institute of Oceanology, Chinese Academy of Sciences 74718, Guangzhou, China; 3 Center for Evolution and Conservation Biology, Southern Marine Sciences and Engineering Guangdong Laboratory (Guangzhou) 606379 , Guangzhou, China; 4 State Key Laboratory of Lake Science and Environment, Nanjing Institute of Geography and Limnology, Chinese Academy of Sciences 66289, Nanjing, China; University of Mississippi, University, Mississippi, USA

**Keywords:** cold seep, microbial diversity, depth variability, community assembly processes

## Abstract

Haima Cold Seep is an active cold seep system in the South China Sea with a thriving biological community that plays an important role in the global carbon cycle. Despite its ecological importance, we know little about the variability of its microbial communities and depth-related structure. In this study, we comprehensively investigated the microbial depth variability and its underlying assembly process in the seepage and non-seepage areas of Haima Cold Seep. We found significant inter-group differences in archaeal (ACC) and bacterial (BCC) other than eukaryotic (ECC) community compositions, between the seepage and non-seepage areas. The seepage area had significantly more archaeal and bacterial 16S rRNA gene copies than the non-seepage area and showed greater fluctuation with depth. Anaerobic methanotrophic archaea (primarily, ANME-3) and sulfate-reducing bacteria (primarily, SEEP-SRB1) were predominant in the seepage area, suggesting that ANME-3 might work in conjunction with sulfate-reducing microorganisms to support the carbon cycle in the cold seep environment. Moreover, there were more lineages found only in specific depth ranges, supporting higher depth variability of both ACC and BCC in the seepage area than in the non-seepage area. The greater depth variability of ACC and BCC in the seepage area appeared to be primarily driven by stronger homogeneous selection imposed by environmental factors (e.g., ammonium). By contrast, eukaryotic community assembly was influenced by random processes (primarily drift) and exhibited no depth dependence. Our findings may help broaden our understanding of the cold seep ecosystem and thus provide clues for its resource utilization.

**IMPORTANCE:**

Marine cold seeps are characterized by the discharge of hydrocarbons and reducing fluids. Rising geological fluids in cold seeps may act as physical transport vectors for deep biosphere microorganisms from the subsurface environment to the surface environment, and thus may influence the depth patterns of microbial community assembly. Despite the ecological importance of microbial communities in cold seeps, we have limited knowledge about their responses to environmental changes along sediment depth and the underlying processes driving these responses. Our study showed that compared with non-seepage area, seepage area exhibited stronger homogeneous selection on prokaryotic community assembly, had more depth-related specialized microorganisms, and supported higher depth variability of both archaeal and bacterial communities. Our findings may provide a theoretical basis for protection and resource utilization of the cold seep ecosystem.

## INTRODUCTION

Marine cold seeps located on continental margins are often accompanied by the discharge of hydrocarbons and reducing fluids (1, 2). The special environment and rich biological communities in cold seeps are of great significance in promoting biogeochemical cycle processes, including those of methane, sulfur, metal elements, and nutrients (3–7). Hydrocarbon-rich fluids (primarily methane) and reducing fluids support rich microbial and animal communities, such as those of anaerobic methanotrophic archaea (ANME) and sulfate-reducing bacteria (SRB), mussels, and other large animals (8–11). The sediment ecosystem in the cold seep acts as a natural barrier to reduce methane released to the overlying water column. Microorganisms use fluids containing methane and sulfate to produce metabolites and energy to support their growth, while various animal communities gain energy through predation and/or through symbiotic relationships with chemotrophic organisms. The collaboration of microorganisms and animals in this ecosystem increases methane oxidation and total oxygen consumption, reducing methane release (12–15). Despite the important ecological functions of the ecosystem, our knowledge is far from comprehensive about the response of the assembly of microbial communities to environmental changes and their underlying mechanisms along sediment depth in the cold seep.

Understanding the assembly mechanism of microbial communities in cold seeps has been a long-standing challenge (16, 17). Microbial community assembly is influenced by both deterministic and random processes (18, 19). Deterministic processes, as defined by niche theory, include interspecies interactions and environmental filtering (20). Random processes include random birth, death, diffusion, extinction, and speciation (21). Deterministic and stochastic processes together affect microbial community assembly, so it is important to understand how their relative contributions vary with heterogeneous environments. Sediment depth may correlate with historical events, mineralogical conditions, and environmental conditions, including nutrient availability and the food web (22–24). Microorganisms are usually sensitive to these abiotic and biotic variables, so microbial diversity and community composition may change with sediment depth (25, 26). The distance decay relationship (DDR), which is based on a mathematical model of the spatial distribution pattern of microorganisms, exists in most prokaryotic and eukaryotic communities in deep-sea ecosystems (27–29). However, the structure changes in prokaryotic and eukaryotic communities with depth in heterogeneous habitats of the cold seeps (e.g., the seepage and non-seepage areas) have seldom been studied (30, 31). In recent years, research on the microbial communities of the cold seeps has mainly focused on a single horizontal and/or vertical scale in shallow sediments (32–34). Significant differences in microbial community compositions were detected between the seepage and non-seepage areas of the cold seep, especially the microorganisms related to the production and consumption of methane, but there are few studies on changes in microbial communities along a depth gradient (35, 36). Recent studies have suggested that rising geological fluids may act as physical transport vectors for deep biosphere microorganisms from the subsurface environment to the surface environment, thereby influencing microbial communities (37). Therefore, understanding the changes with depth, environmental response, and underlying assembly process of microbial communities in heterogeneous habitats of the cold seeps may help us better understand the cold seep ecosystem, and thus provide a theoretical basis for the protection and resource utilization of the cold seep ecosystem.

Haima Cold Seep is an active cold seep system recently discovered in the South China Sea, located in the southern part of the Qiongdongnan Basin (38, 39). To determine how habitat heterogeneity affects microbial communities, we compared microbial communities and environmental factors in sediment cores from seepage and non-seepage areas in the Haima Cold Seep. We use 16S rRNA gene amplicon sequencing to study archaeal (ACC) and bacterial community compositions (BCC), and 18S rRNA gene amplicon sequencing to study eukaryotic community compositions (ECC). We aimed to understand (i) whether there are inter-group differences in the microbial composition between seepage and non-seepage areas of the cold seep, (ii) the differences in changes in microbial community structure with depth and their environmental dependence, and (iii) the underlying assembly mechanisms of microbial communities in the Haima Cold Seep.

## MATERIALS AND METHODS

### Study area, sample collection and environment factor analyses

**Fig 1:**
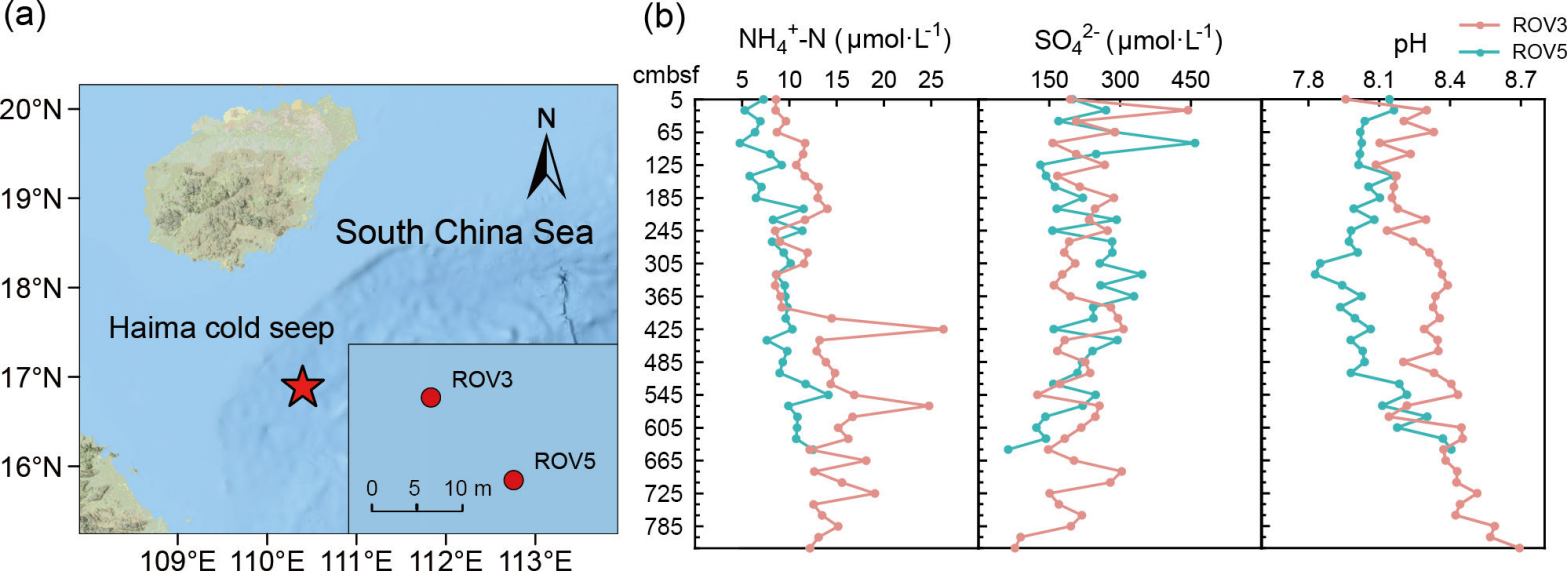
The sampling location (**a**) and the key environmental characteristics (**b**) of the seepage (ROV5) and non-seepage (ROV3) cores of Haima Cold Seep in the South China Sea. The map was generated using open-access online map services in ArcGIS Pro, with data sourced from Esri, NASA, NGA, USGS, TomTom, Garmin, Foursquare, FAO, and METI/NASA.

The two sediment cores were collected with a gravity piston corer from the seepage and non-seepage areas of the Haima Cold Seep in the South China Sea (16.9° N, 110.4° E) (Fig. 1), in September 2020. The seepage (ROV5) and non-seepage (ROV3) areas were identified based on continuous bubbles rising from the seabed and a large number of mussels, which were directly observed using remotely operated vehicles (ROVs) during the sampling. ROV3 was 8.25 m long, and ROV5 was 6.45 m long. The distance between the seepage (ROV5) and non-seepage (ROV3) areas was about 10 m. The cores were sliced into samples (about 5 cm per sample) on board with a 20 cm interval between samples. In addition, pore waters were collected using Rhizons samplers (The Netherlands), which were inserted into the pre-drilled holes in PVC pipes and attached to vacuum tubes by 23G needles under N_2_ atmosphere (40). The samples were immediately frozen in liquid nitrogen and then stored at −80°C before use. Conductivity, pH, and nutrient concentrations (SO_4_^2−^, SiO_3_^2−^-Si, soluble reactive phosphorus [SRP], NH_4_^+^-N, NO_3_^−^-N, and NO_2_^−^-N) of the samples were measured from the pore waters. Conductivity and pH were measured using the Advanced Electrochemistry Meter (ORION VERSASTAR, Thermo Scientific, USA). The concentration of SO_4_^2−^ was measured using the ICS-5000 ion chromatography system (Thermo Scientific, USA), and the concentrations of SiO_3_^2−^-Si, SRP, NH_4_^+^-N, NO_3_^−^-N, and NO_2_^−^-N were measured using an AA3 Auto-analyzer (Seal, Norderstedt, Germany). To measure the concentrations of total carbon (TC), total inorganic carbon (TIC), total nitrogen (TN), and elements, sediment samples were freeze-dried, then ground and homogenized using a mortar and pestle. TC, TIC, and TN were measured using an EA3000 elemental analyzer (Euro Vector, Italy). Metal ion concentrations (Mg^2+^, Al^3+^, K^+^, Ca^2+^, V^4+^, Cr^3+^, Mn^2+^, Fe^2+^, Co^2+^, Ni^2+^, Cu^2+^, Zn^2+^, As^3+^, Se^2+^, Mo^6+^, Ag^+^, Cd^2+^, Sb^3+^, Ba^2+^, Tl^+^, Pb^2+^, Th^4+^, U^3+^, Sc^3+^, Y^3+^, La^3+^, Ce^3+^, Pr^3+^, Nd^3+^, Sm^3+^, Eu^2+^, Gd^3+^, Tb^3+^, Dy^3+^, Ho^3+^, Er^3+^, Tm^3+^, Yb^2+^, and Lu^3+^) were measured using inductively coupled plasma-mass spectroscopy (ICP-MS, NexION 350X, PerkinElmer Inc., Waltham, MA, USA).

### DNA extraction, amplification, sequencing, and data processing

Genomic DNA from each sediment sample (~2 g) was extracted using the PowerSoil DNA Isolation Kit (QIAGEN, Hilden, Germany) by following the manufacturer’s protocol. The extracted DNA was quantified using a NanoDrop 2000 spectrophotometer (Thermo Scientific, Wilmington, DE), with the DNA concentration ranging from 7.85 to 59.40 ng·μL^−1^. The bacterial and archaeal abundance of all samples was determined by quantifying the copy number of the 16S rRNA gene using real-time PCR (qPCR), following the methods outlined in our previous study (41). Bacterial abundance was measured using primers EUB338/EUB518 (42), while archaeal abundance was measured using primers Arch349F/Arch806R (43).

For 16S rRNA gene amplicon sequencing, the V4 region of the 16S rRNA gene was amplified by primers 515FmodF (5′-GTGCYAGCMGCCGCGGTAA-3′) and 806RmodR (3′-GGACTACNVGGGTWTCTAAT-5′) using the Illumina HiSeq PE250 platform. The 18S rRNA gene was amplified using primers 528F(5ʹ-GCGGTAATTCCAGCTCCAA-3ʹ) and 706R (5ʹ-AATCCRAGAATTTCACCTCT-3ʹ). Three replicates of each sample were PCR amplified in a 50 µL reaction mixture consisting of 25 µL 2× PCR Premix Taq, 10 mM for each primer, 60 ng genomic DNA, and 20 µL nuclease-free water. The DNA of each sample was amplified using the following conditions: initial denaturation at 95°C for 5 minutes; 35 cycles including 95°C denaturation for 45 seconds, 56°C annealing for 1 minute, 72°C extension for 1 minute, and finally extending at 72°C for 10 minutes. The PCR products from the three replicates were purified, combined, and sequenced on the Illumina HiSeq 2500 platform at BGI (Shenzhen, China). The obtained raw sequences were analyzed using the mothur package (http://www.mothur.org). Briefly, the original sequences were combined, de-noised, trimmed, quality filtered, and compared against the SILVA v138 database (http://www.arb-silva.de/) using mothur (v.1.36.1，http://www.mothur.org). The lineages belonging to chloroplasts and mitochondria were removed, and after preliminary treatment, the high-quality sequences were clustered into ASVs (Amplicon Sequence Variants) (44). High-quality sequences were further classified using the SILVA v138 databases at a recommended bootstrap threshold of 80%. To minimize bias of sequencing depth, ASVs that appeared in fewer than two samples were excluded. We totally obtained 10,007 ASVs for prokaryotic sample sets and 1,020 ASVs for eukaryotic sample sets. The minimum number of sequences (31,217 for prokaryotic sample sets and 12,796 for eukaryotic sample sets) was randomly resampled in the whole sample set to correct the differences in sequencing depth. In this study, “the relative abundance of certain microbial taxa for 16S/18S rRNA genes” was abbreviated to “the relative abundance of certain microbial taxa.”

### Microbial community phylogenetic structure and assembly process

The ClustalW algorithm was used in MEGA 11 to compare the representative sequences of the selected ASVs with the reference sequences (45) retrieved from the National Center for Biotechnology Information (NCBI) (https://www.ncbi.nlm.nih.gov/), and we reconstructed the phylogenetic tree using the neighbor-joining method in MEGA 11 (46). Community phylogenetic structure analysis was examined to uncover microbial community assembly patterns and their possible underlying causes along depth gradients. The phylogenetic diversity (PD) and the standardized-effect size of mean nearest taxon distance (SES.MNTD) were calculated using the pd and ses.mntd in the “picante” package in R, respectively (47). In addition, we analyzed the community assembly mechanism using the phylogenetic bin-based null model analysis (iCAMP) framework (48). The iCAMP was used to quantify the relative importance of different ecological processes. The highest possible number of bins is jointly determined by the values of *d_s_* and *n*_min_, and we use the empirical value *d_s_* = 0.2, which is a phylogenetic signal threshold proven to robustly discern microbial niche preferences (49, 50). We determined the *n*_min_ value by performing a series of *n*_min_ value tests on the model (*n*_min_ = 24 in this study).

### Calculation and division of specialized ASVs

To examine the distributions of ASVs in two different habitats and their habitat specificity, the specific ASVs in different habitats were calculated according to the method of Dufrene and Legendre (51). In the following formula, the specificity of ASVs in habitat H is the ratio of the average abundance of S in all H samples (${Nindividuals}_{S,H}$) to the sum of the average abundance of S in all habitats in this study (${Nindividuals}_{S}$). Among all samples where ASVs exist in habitat H, the ratio of the number of samples of S (${Nsites}_{S,H}$) to the number of samples of H (${Nsites}_{H}$) is H.


(1) $$Specificity=\frac{{\text{ Nindividuals }}_{S,H}}{{\text{ Nindividuals }}_{S}}\#$$

(2) $$Occupancy=\frac{{\text{ Nsites }}_{S,H}}{{\text{ Nsites }}_{H}}\#$$

Specialized ASVs are habitat-specific and ubiquitous in most locations in the same habitat. To identify specialized ASVs in each habitat, ASVs with a specificity (equation 1) and occupancy (equation 2) ≥0.7 are selected as specialized species, while the rest are non-specialized species (52).

### Statistical analyses

Microbial diversity was calculated using the vegan package in R. The alpha diversity was estimated as the number of ASVs (richness), Shannon index, and Faith’s phylogenetic diversity (PD) of each sample. Non-metric multidimensional scaling (NMDS) and β-diversity hierarchical clustering based on Bray-Curtis’s distance were used to analyze the distribution pattern of microbial communities, and a general additive model was established using depth and ranking axes to observe the position of sample sites on the depth gradient. Analysis of similarities (ANOSIM), multi-response permutation procedure (MRPP), and permutational multivariate analysis of variance (PERMANOVA) were used to test for significant differences in microbial communities between seepage (ROV5) cold seep and non-seepage (ROV3) one. Since the length of the first detrended correspondence analysis (DCA) axis was >4, canonical correspondence analysis (CCA) was used to examine the relationships between microbial communities and environmental parameters, which was performed using the vegan package in R. Before the CCA, we conducted forward selection through the “ordiR2step” automatic selection program in the vegan package in R to determine the best subset of explanatory variables. We used MRM to analyze the correlations between environmental factors and community structure, and the variation of Bray–Curtis dissimilarity was divided into (i) depth variation, (ii) variation in pure metals, (iii) other environmental variation, and (iv) unexplained variation using the ecodist package in R. Due to the strong collinearity among specific environmental factors, we used variable clustering through the varclus procedure in the Hmisc package of R to assess the redundancy of environmental variables, removing more correlated variables from the MRM analysis (Spearman ρ^2^ > 0.7). To find the key environmental variables, the pure effect of the environment was further tested via partial Mantel tests using the mantel.partial command in the vegan package in R. The psych package in R was used to depict the Spearman correlations between the specialized species and environmental factors of the most abundant 29 ASVs. Linear fitting regression analysis was performed to test the relationships between each parameter and the sediment depth.

## RESULTS

### Environmental parameters in the seepage and non-seepage areas of the cold seep

Among the measured environmental factors, SRP, NH_4_^+^-N, pH, conductivity, Mg^2+^, Al^3+^, and Ba^2+^ in the non-seepage area were significantly higher than those in the seepage area, while Mn^2+^ and Mo^6+^ in the seepage area were higher than those in the non-seepage area (*P* < 0.01 in all cases; Fig. S1). It was worth noting that there was no significant difference in SO_4_^2−^ concentration between non-seepage (77.30–443.71 μmol·L^−1^) and seepage (63.12–458.46μmol·L^−1^) areas (Fig. 1), and we found that the NH_4_^+^-N in the non-seepage area varied greatly with depth (Fig. 1). For pH, it first decreased with depth and then increased, reaching the lowest pH at a depth of 3.25 m in the seepage area (Fig. 1). In addition, some soil environmental properties showed significant differences across depth gradients. In the non-seepage area, SO_4_^2−^ (*r* = 0.36, *P* < 0.05), TN (*r* = 0.35, *P* < 0.05), and U^3+^ (*r* = 0.40, *P* < 0.01) significantly decreased with increasing depth, while SRP (*r* = 0.41, *P* < 0.01), NH_4_^+^-N (*r* = 0.49, *P* < 0.001), pH (*r* = 0.78, *P* < 0.001), Mg^2+^ (*r* = 0.60, *P* < 0.001), K^+^ (*r* = 0.34, *P* < 0.05), Mo^6+^ (*r* = 0.41, *P* < 0.01), and Tl^2+^ (*r* = 0.40, *P* < 0.01) increased significantly with increasing depth (Table S1). In the seepage area, TN (*r* = 0.51, *P* < 0.01), Mo^6+^ (*r* = 0.61, *P* < 0.001), and Sb^2+^ (*r* = 0.51, *P* < 0.01) significantly decreased with increasing depth, while NH_4_^+^-N (*r* = 0.74, *P* < 0.001), pH (*r* = 0.39, *P* < 0.05), Mg^2+^ (*r* = 0.54, *P* < 0.01), K^+^ (*r* = 0.37, *P* < 0.05), and Mn^2+^ (*r* = 0.55, *P* < 0.001) were the opposite (Table S1). Among them, NH_4_^+^-N, TN, pH, Mg^2+^, K^+^, and Mo^6+^ were the most sensitive to changes with depth in both seepage and non-seepage areas of the cold seep.

### Microbial community compositions in the seepage and non-seepage areas of the cold seep

**Fig 2:**
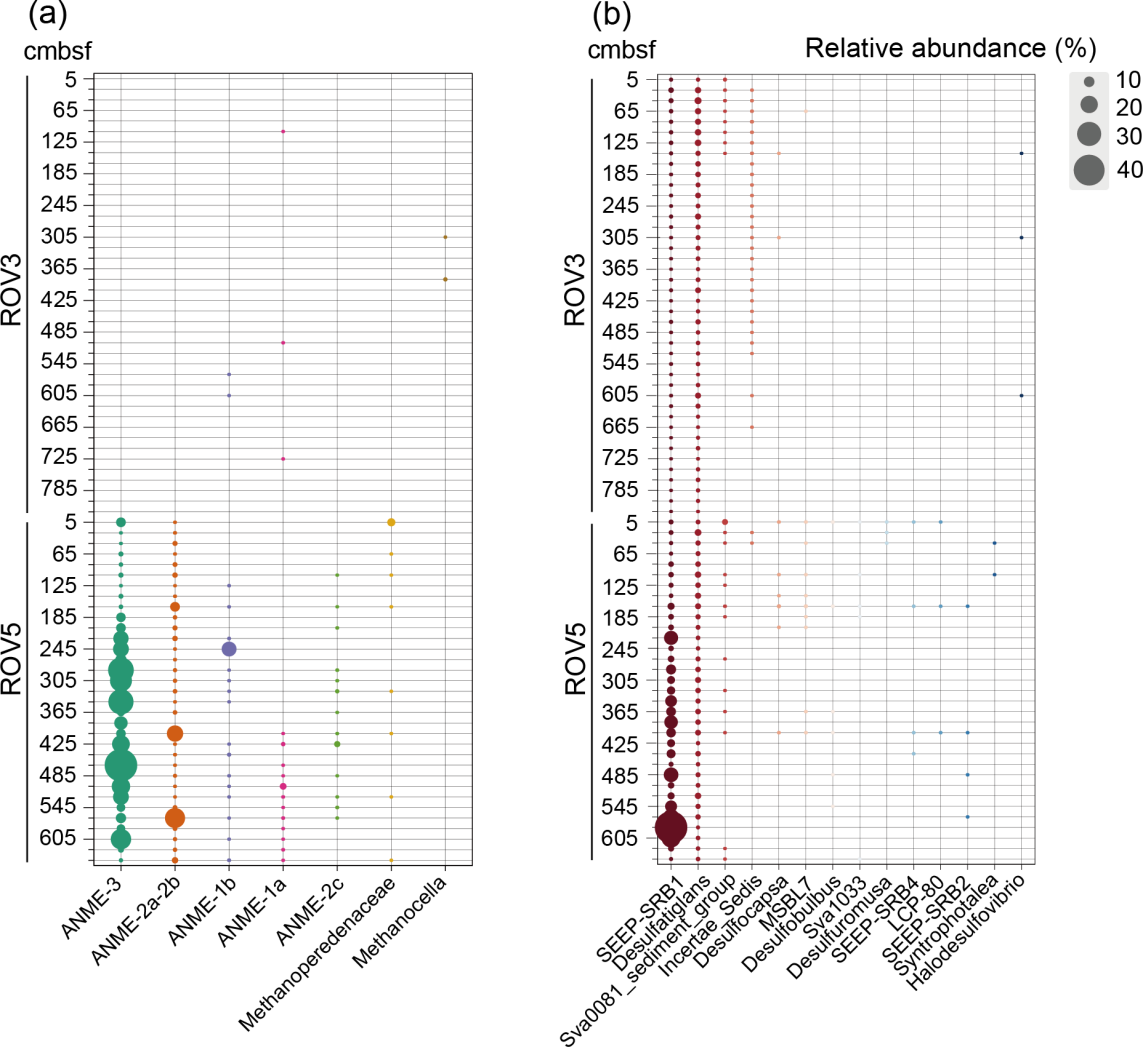
The relative abundances of key functional microbial groups, including ANME, methanogens, and SRB, were analyzed in seepage (ROV5) and non-seepage (ROV3) areas of the cold seep, alongside the phylogenetic relationships of ANME and methanogens. (**a**) The relative abundances of dominant subgroups of ANME and methanogens within the phylum Halobacterota. (**b**) The relative abundances of dominant subgroups of SRB within the phylum Desulfobacterota.

Although the overall abundance of archaeal and bacterial 16S rRNA genes in the seepage area of the cold seep was significantly higher than in the non-seepage area (Wilcoxon test, *P*  <  0.05), their abundance exhibited greater fluctuations with depth (Fig. S2). The dominant groups of the seepage and non-seepage areas were different. For ACC, Crenarchaeota (8.26%–79.54% for non-seepage area; 0.51%–56.76% for seepage area), Asgardarchaeota (1.36%–37.30% for non-seepage area; 7.93%–35.71% for seepage area), and Nanoarchaeota (4.72%–31.40% for non-seepage area; 4.25%–51.82% for seepage area) were dominant groups of both seepage and non-seepage areas (Fig. S3). The relative abundance of Crenarchaeota in the non-seepage area was higher, whereas there was a higher relative abundance of Halobacterota and Nanoarchaeota in the seepage area. As for BCC, Gammaproteobacteria (0.52%–79.86% for non-seepage area; 1.22%–77.16% for seepage area), Chloroflexi (7.59%–49.31% for non-seepage area; 4.20%–30.51% for seepage area), and Caldatribacteriota (0.00%–15.38% for non-seepage area; 0.93%–46.79% for seepage area) were dominant groups in both the seepage and non-seepage areas (Fig. S3). It is worth noting that the relative abundance of Caldatribacteriota (0.00%–15.38% for non-seepage area; 0.93%–46.79% for seepage area) and Desulfobacterota (0.40%–8.86% for non-seepage area; 2.07%–46.04% for seepage area) was much higher in the seepage area than in the non-seepage area (Fig. S3). As for the ECC, Ascomycota (0.43%–36.75% for non-seepage area; 1.56%–87.07% for seepage area), Cercozoa (0.00%–37.93% for non-seepage area; 0.77%–14.29% for seepage area), and Protalveolata (0.00%–37.82% for non-seepage area; 0.00%–43.33% for seepage area) were the dominant groups in the two areas of the cold seep (Fig. S3). The relative abundances of some of the above microbes were also correlated with other environmental parameters, including pH, metals, and nutrient concentrations (Table S2). The NH_4_^+^-N and pH were correlated with the relative abundance of several major phyla, and it should be noted that they were all negatively correlated with Halobacterota, Caldatribacteriota, and Desulfobacterota (*P*  <  0.05 in all cases; Table S2). At the clade level, the distribution of microorganisms involved in methane and sulfur metabolism was analyzed. ANME were more abundant at most depths in the seepage area (0.00%–45.43%) and almost not detected in the non-seepage area (0.00%–0.22%, Fig. 2). In the seepage area, a higher abundance of ANME-3 (0.25%–45.43%) was observed compared to other groups such as ANME-2a-2b (0.00%–25.74%), ANME-1b (0.00%–17.47%), ANME-1a (0.00%–4.70%), and ANME-2c (0.00%–3.83%, Fig. 2). We constructed a neighbor-joining phylogenetic tree to show phylogenetic relationships between ANME and methanogenic 16S rRNA gene sequences. The results showed that the 4 ASVs had high similarity with sequences associated with the ANME-3 cluster (Fig. S4). Moreover, both ANME-2 (i.e., ANME-2c, ANME-2b, and ANME-2a-2b) and ANME-3 show a relatively distant phylogenetic relationship with the clade of ANME-1 (i.e., ANME-1a and ANME-1b) (Fig. S4). For SRB, SEEP-SRB1 was abundant at most depths in the seepage area (0.62%–42.49%) but was not prevalent in the non-seepage area (0.04%–2.63%) (Fig. 2). It is noteworthy that we observed significant positive correlations between the relative abundance of ANME-3 and ANME-2c with that of SEEP-SRB1, respectively (*P* < 0.05) (Fig. S5). In addition, 66 and 29 specialized ASVs were selected in non-seepage and seepage areas, respectively. Among the most abundant 29 specialized ASVs, the relative abundance of most of the specialized ASVs in the seepage area showed significant correlations with depth (*P*  <  0.05; Fig. S6). In addition, most of the specialized ASVs in the seepage area were also significantly correlated with NH_4_^+^-N, Mn^2+^, and Mo^6+^ (*P* < 0.05 in all cases; Fig. S6).

### Diversity of microbial communities in seepage and non-seepage areas of the cold seep

**Fig 3:**
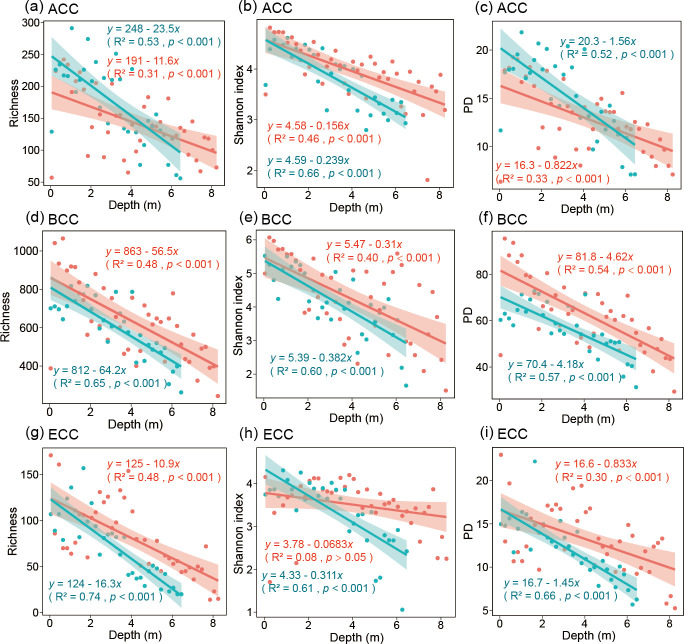
Linear correlation analysis of alpha-diversity indices (Shannon, richness, and PD index) and depth of ACC (a–c), BCC (d–f), and ECC (g–i) in seepage (ROV5, blue) and non-seepage (ROV3, red) areas.

The richness and Shannon indices of ACC, BCC, PCC (prokaryotic community composition), and ECC in the seepage area showed a greater variation rate with depth than those in the non-seepage area. For ACC, the richness and PD indices in the seepage area were significantly higher than those in the non-seepage area (*t* test, *P* < 0.05 in all cases), but there was no significant difference in the Shannon index (*t* test, *P* > 0.05) (Fig. S7). For BCC, PCC, and ECC, there was no significant difference in alpha diversity between the seepage and non-seepage areas (*t* test, *P* > 0.05 in all cases; Fig. S7 and S8). To explore the variation trend with depth, we fitted them linearly. Whether for ACC, BCC, or PCC, there were significant decreases in Shannon, richness, and PD indices toward increasing depth (*P* < 0.001 in all cases; Fig. 3; Fig. S8). For ECC, we found that the richness (non-seepage area: *r*^2 ^= 0.48, *P <* 0.001; seepage area: *r*^2 ^= 0.74, *P <* 0.001) and PD indices (non-seepage area: *r*^2 ^= 0.30, *P <* 0.001; seepage area: *r*^2 ^= 0.66, *P <* 0.001) decreased with increasing depth (Fig. 3). For the ECC Shannon index, it decreased with increasing depth in the seepage area (non-seepage area: *r*^2 ^= 0.08, *P >* 0.05; seepage area: *r*^2 ^= 0.61, *P <* 0.001), but not in the non-seepage area (Fig. 3). In addition, for seepage area, there was a negative correlation between NH_4_^+^-N and microbial alpha diversity, while there was a positive correlation between total Mo^6+^ and microbial alpha diversity (*P*  <  0.01 in all cases; Fig. S9).

### Relating community structure to environmental factors

**Fig 4:**
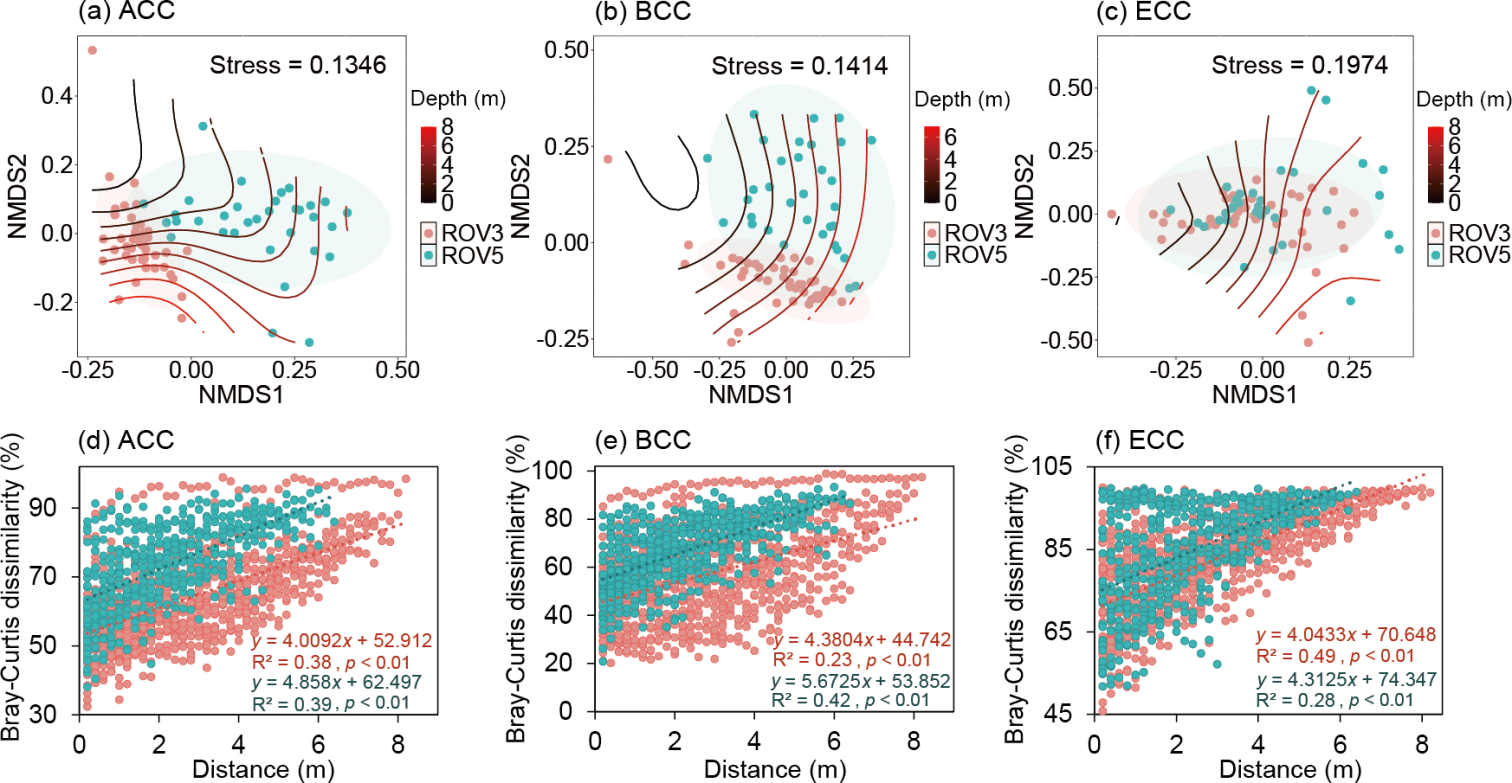
NMDS ordinations based on the Bray–Curtis dissimilarity of ACC (**a**), BCC (**b**), and ECC (**c**), and the relationships between depth distance and dissimilarities of ACC (**d**), BCC (**e**), and ECC (**f**) in seepage (ROV5, blue) and non-seepage (ROV3, red) areas.

NMDS suggested ACC, BCC, and PCC of seepage and non-seepage areas were distinctly separated (*P*  <  0.01 in all cases), while ECC was not (*P*  >  0.05 in all cases; Fig. 4; Table S3). We observed that the beta diversity of all microbial communities (ACC, BCC, PCC, and ECC) in the seepage area was higher than that in the non-seepage area (*P* <  0.001 in all cases; Fig. S10), and the community differences of them were positively correlated with the differences in depth (*P* < 0.01; Fig. 4; Fig. S11), indicating a close link between depth and microbial community structure.

**Fig 5:**
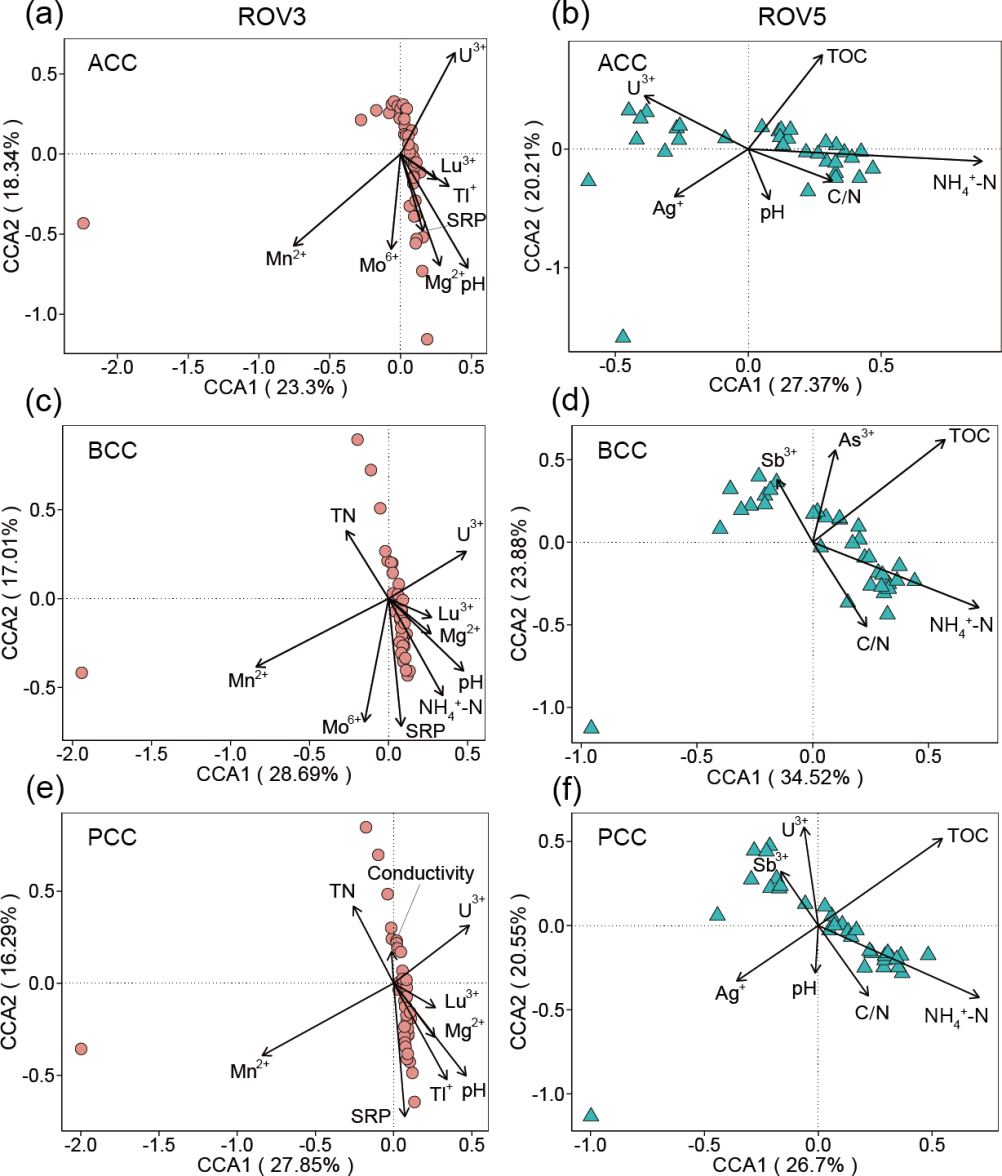
Canonical correspondence analysis (CCA) relating environmental variables to archaeal (ACC; a and b), bacterial (BCC; c and d), and prokaryotic community compositions (PCC; e and f) in seepage (ROV5; b, d and f) and non-seepage (ROV3; a, c and e) areas.

Depth was a significant factor affecting the microbial community compositions (i.e., ACC, BCC, and PCC) of both seepage and non-seepage areas, as shown by the results of the partial Mantel test (*P* < 0.001 in all cases; Tables S4 and S5). Multiple regression on dissimilarity matrices (MRM) showed that for the overall PCC, environmental variables explained 46.0% in non-seepage areas but 51.8% in seepage ones (Fig. S12). For ACC and BCC, environmental variables explained 66.5% and 42.8% in the non-seepage area (Fig. S13), respectively, but explained 43.0% and 49.2% in the seepage one (Fig. S13). It was worth noting that all the effects of pure depth distance on microbial community dissimilarity in the seepage area (33.7% for ACC; 23.9% for BCC; 30.9%% for PCC) were higher than that in the non-seepage area (15.5% for ACC; 14.1% for BCC; and 15.4% for PCC). In addition, partial Mantel test results showed that ACC, BCC, and PCC of the non-seepage area were significantly correlated with pH and Mn^2+^, while those of the seepage area were significantly correlated with NH_4_^+^-N and TOC (*P* < 0.05 in all cases). These results were also supported by CCA (*P* < 0.01 in all cases) (Fig. 5; Tables S4 and S5).

### Microbial community assembly processes in seepage and non-seepage areas of the cold seep

**Fig 6:**
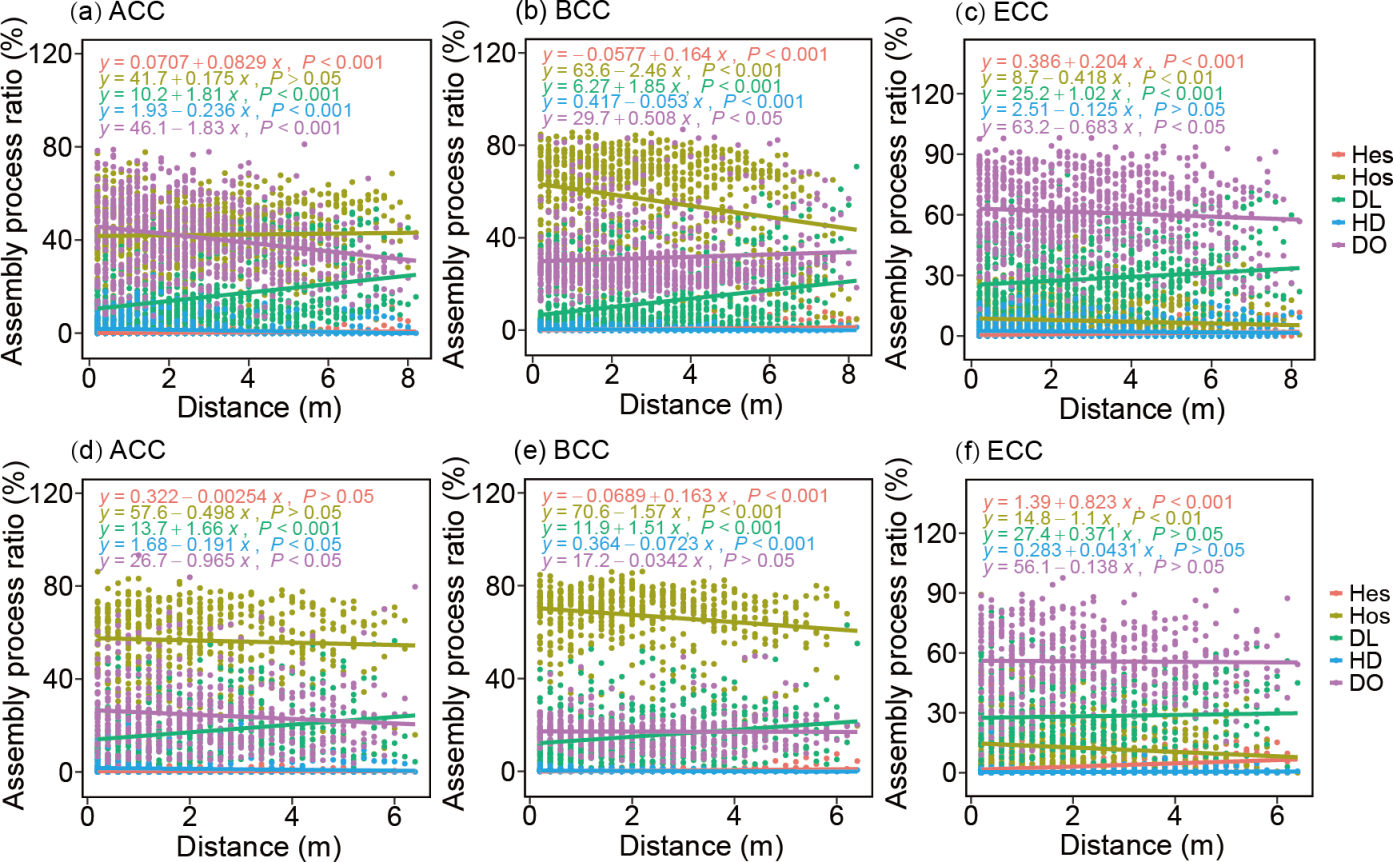
Linear regression between distance and the relative percentage of different community assembly processes of ACC (**a**), BCC (**b**), and ECC (**c**) in seepage (ROV5) and ACC (**c**), BCC (**d**), and ECC (**f**) in non-seepage (ROV3) areas. Different colors represent different assembly processes. HeS, heterogeneous selection; HoS, homogeneous selection; DL, dispersal limitation; HD, homogenizing dispersal; DR, drift and others.

To understand the assembly mechanism of microbial communities, we conducted a phylogenetic framework analysis and found that the standardized-effect size of the MNTD (SES.MNTD) of PCC for both seepage and non-seepage areas was significantly lower than zero and increased linearly with depth, suggesting that prokaryotic communities were more phylogenetically clustered via environmental filtering than expected at random (*P* < 0.001 in all cases; Fig. S14). It is worth noting that SES.MNTD of the seepage area had a higher rate of change with depth than the non-seepage one (Fig. S14 and S15). This finding was further confirmed by the phylogenetic null model. Our results showed that for BCC, both seepage and non-seepage areas were dominated by deterministic assembly, primarily homogeneous selection. Moreover, homogeneous selection had a greater effect on BCC in the seepage area (67.02%) than on the non-seepage one (56.58%) (Permutation *t* test: *P*  <  0.001; Fig. S16). Conversely, drift, which represents random changes in microbial community composition due to stochastic processes, such as random births, deaths, and events, had a greater impact on the PCC assembly process in the non-seepage area (35.83%) compared to the seepage one (16.97%) (permutation *t* test: *t*  = −24.249, *P*  <  0.001; Fig. S17). For ECC, stochastic assembly, primarily drift and other factors, dominated community assembly for both non-seepage (61.24%) and seepage (55.80%) areas (Fig. S16). We correlated each of the assembly processes with the depth distance, and the results showed that for both ACC and BCC, the relative importance of homogeneous selection remained higher throughout the depth profile in the seepage area than in the non-seepage one (Fig. 6). It is worth noting that for ACC, BCC, and PCC, the dispersal limitations of seepage and non-seepage areas were significantly positively correlated with depth (*P*  <  0.001 in all cases), and the change rate of non-seepage area with the depth distance was higher than that of the seepage one (Fig. 6; Fig. S17).

## DISCUSSION

### Significant differences in microbial community compositions between seepage and non-seepage areas of the cold seep

The main archaeal phyla, such as Crenarchaeota, Asgardarchaeota, and Nanoarchaeota, were detected in both habitats. They are often found in cold seep sediments, and Asgardarchaeota are involved in the carbon cycle (53–57). As for bacteria, Gammaproteobacteria, Chloroflexi, and Caldatribacteriota were the main bacterial groups detected in both the seepage and non-seepage samples, consistent with other marine sediment studies (58–62). They are an important part of the carbon and sulfur cycles in the ocean. Caldatribacteriota, Desulfobacterota, and Halobacterota were detected at higher relative abundance in the seepage area, which is also consistent with other studies (63, 64). Compared to the non-seepage area, there was a weaker alkaline environment and lower ammonia nitrogen levels in the seepage one. Environmental factors typically act as “filters” to select for groups that are adapted to these environmental characteristics, thereby promoting changes in the relative abundance of dominant groups (65). The decrease in ammonia nitrogen content in the seepage area may restrict the nitrogen source, which, in turn, restricts some ammonia-dependent growth conditions and promotes the nitrogen-fixing microbial groups. Caldatribacteriota have been found to have the potential for nitrogen fixation, which is particularly important in the nutrient-poor deep-sea environment, and the ability to fix nitrogen can help these microbial groups grow in low ammonia nitrogen environments while providing a new source of nitrogen for the ecosystem (66). Other studies have pointed out that some groups of Caldatribacteriota have the potential to provide substrate for methanogenesis through sugar and amino acid fermentation, and they may interact with methanogens to improve the survival rate, which may be the reason for their dominance in the seepage area (67). Desulfobacterota and Halobacterota are major players involved in the anaerobic oxidation of methane and sulfate reduction (68–73), and studies have indicated that the microbial groups involved in this process are more active in low-pH environments (74). In the seepage area, we observed a higher abundance of ANME and SRB, which is consistent with reports from other studies (75, 76). In our study, ANME (ANME-1,-2, and -3) and SRB were highly enriched in the seepage area. The distribution of different branches of ANME was diverse. ANME1 mainly occurs in shallow layers and sedimentary zones that are devoid of sulfate. ANME-2 tends to be the primary component of the surface layer, while ANME-3 is found in mud volcanoes and is typically enriched in deep cold seep sediments with high sulfate concentrations (77–79). ANME-1 and ANME-2 are widely distributed in cold seep sediments around the world, while ANME-3 is extremely rare (7, 36). This study found that the relative abundance of ANME-3 was significantly higher than that of ANME-1 and ANME-2, highlighting the potential selection of environmental differences (e.g., variations in methane supply) on microbial community composition across different habitats. Similar observations have been reported in recent studies, such as those from the Haima Cold Seep in the South China Sea (32) and shallow cold seeps in the northern South China Sea (63). These findings suggest that ANME-3 is a dominant group within methane-metabolizing communities, potentially co-contributing to the carbon cycle in cold seep environments alongside sulfate-reducing microorganisms (32, 63). Our results revealed significant correlations between SEEP-SRB1 and both ANME-3 and ANME-2c. These findings suggest potential associations between SEEP-SRB1 and ANME-3, as well as SEEP-SRB1 and ANME-2c. These potential associations were further confirmed by a previous study (80), which shows that SEEP-SRB1 can also couple with ANME-3, using catalyzed reporter deposition-fluorescence *in situ* hybridization (CARD-FISH). It indicates that SEEP-SRB1 may also be the partner of ANME-3 aggregates and carry out SO_4_^2–^-AOM together.

### The seepage area of the cold seep supported higher depth variability of both archaeal and bacterial community compositions than the non-seepage one

The change rate of the alpha diversity indices (i.e., richness and Shannon indices) with depth of the community (i.e., ACC, BCC, PCC, and ECC) of the seepage area was higher than that of the non-seepage one, which indicated that the microbial community compositions in the seepage area changed faster than that of the non-seepage area. The different behavior of PCC in seepage and non-seepage areas might also indicate their different levels of sensitivity to depth changes. Depth is a proxy for numerous environmental factors that may co-vary with changes in many environmental factors. The physicochemical factors vary along sediment depth gradients, such as pH, and concentrations of metallic elements and nutrients (81–83), thus affecting the diversity and distribution of microbial communities (84, 85). In our study, as demonstrated by CCA analysis, the PCC of seepage and non-seepage areas was significantly related to multiple environmental conditions. Microorganisms with wider niches are usually better able to adapt to environmental change and regulate their diversity, composition, and function (86–89). The greater variation rate of community diversity with depth in the seepage area may be related to the fact that there are more depth-related specialized ASVs among the most abundant ASVs, as our results showed. In our study, we found that the diversity of PCC and ECC in the non-seepage area was significantly negatively correlated with pH. This may be because a higher pH environment will filter out more specific microorganisms, thereby reducing the community diversity (90, 91). It has been suggested that microbial communities in extreme environments are less diverse than in more stable, homogeneous environments (92, 93). Different from previous studies, the richness and PD indices of ACC in the seepage area were significantly higher than those in the non-seepage area, indicating that ACC in the seepage area is more abundant and has higher phylogenetic diversity. It has been suggested that more diverse metabolic activities exist in anaerobic methane-oxidizing archaea of the seepage area in the cold seep, which may provide nutrients for the growth and reproduction of archaea, thereby increasing the diversity (11, 79).

### Higher depth variability of PCC in the seepage area of the cold seep was dominantly shaped by stronger homogeneous selection

**Fig 7:**
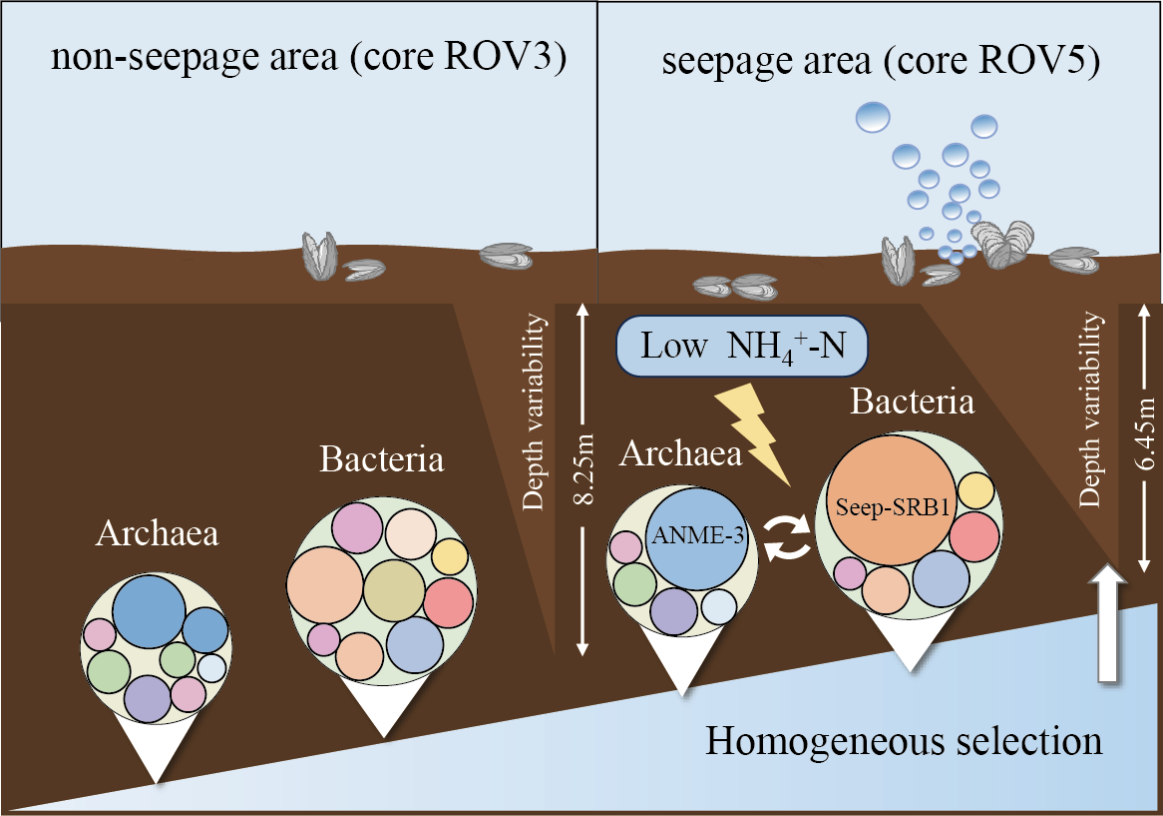
The concept map summarizing the depth variability and assembly processes of prokaryotic communities in non-seepage (ROV3) and seepage (ROV5) areas.

Elucidating the assembly process of microbial communities is important for understanding biodiversity patterns and ecosystem functions (94–97). By comparing the phylogenetic structure of microbial communities, the relative importance of deterministic and stochastic processes in microbial community assembly can be deduced (48, 98). In this study, we found that the standardized effect sizes of MNTD for ACC, BCC, and PCC were significantly below zero and increased with depth, and the seepage area had a higher depth change rate than the non-seepage one. These results suggested that depth was related to the relative importance of deterministic and stochastic processes in the assembly of prokaryotic communities (i.e., ACC, BCC, and PCC) and had a greater effect in the seepage area than the non-seepage. An analysis linking the null model of the phylogenetic structure to depth further confirmed this finding. Our results showed that depth affected prokaryotic communities (i.e., ACC, BCC, and PCC), primarily by influencing dispersal limitation. An important effect of dispersal limitation was that community similarity was expected to decline along a spatial distance gradient (20). In our study, this was confirmed by significant depth patterns of the ACC, BCC, and PCC of seepage and non-seepage areas, suggesting that as the depth distance differences increased, the heterogenization of the microbial communities gradually occurred. The higher depth variability of PCC in the seepage area was mainly shaped by stronger homogeneous selection by gradual changes in environmental factors (e.g., ammonium, sulfate, methane, pH, and redox conditions) across depths, which confirmed previous findings that active seepage enhanced the relative importance of homogeneous selection, mainly by acting on anaerobic methane-oxidizing archaea and sulfate-reducing bacteria involved in anaerobic methane oxidation (35). We found a large number of anaerobic oxidation archaea (primarily ANME-3) and sulfate-reducing bacteria (primarily SEEP-SRB1) in the seepage area, which are involved in methane and sulfate metabolism and play a key role in the carbon cycle of the ecosystem (99–101). In addition, studies have shown that ammonia nitrogen and organic carbon significantly affect microbial biosynthesis, cell growth, and cell cycle progression (102). The low ammonia nitrogen environment in the seepage area may restrict the growth of some microorganisms and accelerate the growth of groups involved in functions, such as methane metabolism, thus strengthening the homogeneous selection. This was evidenced by the relative abundance of Caldatribacteriota, Desulfobacterota, and Halobacterota in the seepage area and their negative correlations with ammonia nitrogen. We synthesized the main findings of our study, as described in the concept map below (Fig. 7). For ECC, the seepage and non-seepage areas were mainly affected by random assembly, mainly drift and other factors. The assembly of communities may be explained by species characteristics (103–105), niche (106), and dispersal limitation (107).

### Conclusion

The results revealed that anaerobic methanotrophic archaea (primarily, ANME-3) and sulfate-reducing bacteria (primarily, SEEP-SRB1) were predominant in the seepage area. This suggests that ANME-3 may represent a major group within the methane-metabolizing community, potentially working in conjunction with sulfate-reducing microorganisms to support the carbon cycle in the cold seep environment. We found that not only did microbial diversity decrease with increasing depth, but community composition also changed significantly. Compared with non-seepage areas, seepage areas exhibited stronger homogeneous selection of environmental factors (e.g., lower ammonium in the seepage area) on prokaryotic community assembly, had more depth-related specialized microorganisms, and supported higher depth variability of both archaeal and bacterial communities. These findings emphasized the importance of seepage fluids in shaping microbial depth patterns and their potential ecological functions in cold seep ecosystems, which may be conducive for understanding methane cycling, ecosystem stability, and the potential for resource utilization in cold seep environments.

## Data Availability

The sequencing data obtained were deposited into the Sequence Read Archive (SRA) of the National Center for Biotechnology Information (https://submit.ncbi.nlm.nih.gov/subs/sra) database under accession numbers PRJNA1131966 (TaxID:749907) and PRJNA1133151 (TaxID:749907). Other environmental data will be made available on request.

## SUPPLEMENTAL MATERIAL

Supplemental tables and figures spectrum.02722-24-s0001.docx Table S1 to S6 and Fig. S1 to S17.

ASM does not own the copyrights to Supplemental Material that may be linked to, or accessed through, an article. The authors have granted ASM a non-exclusive, world-wide license to publish the Supplemental Material files. Please contact the corresponding author directly for reuse.
