# Supplementary material for: Seepage area of the cold seep exhibits strong homogeneous selection on prokaryotic community assembly and supports high depth variability of both archaeal and bacterial communities
Source: Microbiol Spectr. 2025 Jun 10;13(7):e02722-24. doi: 10.1128/spectrum.02722-24 (PMC12210922; doi:10.1128/spectrum.02722-24)
Supplement: Supplemental tables and figures — Table S1 to S6 and Fig. S1 to S17. [file spectrum.02722-24-s0001.docx]

**Supplemental Materials**

**Seepage area of the cold seep exhibits strong homogeneous selection on prokaryotic community assembly and supports high depth variability of both archaeal and bacterial communities**

Xueling Xiong ^a#^, Furun Li ^b#^, Haokun Yang ^a^, Chunshan Li ^b^, Haiming Chen ^a^, Dan He ^c^, Qinglong L. Wu ^c, d^, Sijun Huang ^b *^, Lijuan Ren ^a*^

^a^ Department of Ecology and Institute of Hydrobiology, Jinan University 510610, Guangzhou, China

^b^ CAS Key Laboratory of Tropical Marine Bio-resources and Ecology, South China Sea Institute of Oceanology, Chinese Academy of Sciences 510301, Guangzhou, China

^c^ Center for Evolution and Conservation Biology, Southern Marine Sciences and Engineering Guangdong Laboratory (Guangzhou) 511458, Guangzhou, China

^d^ State Key Laboratory of Lake Science and Environment, Nanjing Institute of Geography and Limnology, Chinese Academy of Sciences 210008, Nanjing, China

E-mail address of each author:

Xueling Xiong: xueling@stu2022.jnu.edu.cn;

Furun Li: lifurun18@mails.ucas.ac.cn;

Haokun Yang: yanghaokun@stu2022.jnu.edu.cn;

Chunshan Li: lichunshan22@mails.ucas.ac.cn;

Sijun Huang: huangsijun@scsio.ac.cn;

Haiming Chen: jnumingmlvs@stu2022.jnu.edu.cn;

Dan He: he_dan@gmlab.ac.cn;

Qinglong L. Wu: qlwu@niglas.ac.cn;

Lijuan Ren: lijuanren@jnu.edu.cn.

^#^ Xueling Xiong and Furun Li contributed equally to this work.

* Address correspondence to Lijuan Ren and Sijun Huang, Email: [lijuanren@jnu.edu.cn](mailto:lijuanren@jnu.edu.cn); and huangsijun@scsio.ac.cn

Running Head: Microbial community assembly in Haima Cold Seep

**Table S1** Linear correlation analysis between environmental variables and depth in the seepage area (ROV5) and non-seepage area (ROV3).

| Environmental factors | ROV3 | |  | ROV5 | |
| --- | --- | --- | --- | --- | --- |
|  | r | p |  | r | p |
| SO_4_^2-^ (μmol·L^-1^) | -0.36 | 0.02* |  | -0.31 | 0.08 |
| Si (μmol·L^-1^) | -0.02 | 0.92 |  | 0.00 | 0.99 |
| SRP (μmol·L^-1^) | 0.41 | 0.01** |  | 0.05 | 0.77 |
| NH_4_^+^-N (μmol·L^-1^) | 0.49 | 0.00*** |  | 0.74 | 0.00*** |
| TN (%) | -0.35 | 0.02* |  | -0.51 | 0.00** |
| TC (%) | -0.17 | 0.29 |  | -0.12 | 0.50 |
| TOC (%) | -0.25 | 0.11 |  | -0.01 | 0.95 |
| TIC (%) | -0.08 | 0.60 |  | -0.12 | 0.49 |
| C/N | 0.05 | 0.77 |  | 0.22 | 0.22 |
| pH | 0.78 | 0.00*** |  | 0.39 | 0.03* |
| Conductivity (mS·cm^-1^) | -0.11 | 0.49 |  | -0.19 | 0.30 |
| Mg^2+^ (μg·g^-1^) | 0.60 | 0.00*** |  | 0.54 | 0.00** |
| Al^3+^ (μg·g^-1^) | -0.06 | 0.71 |  | 0.05 | 0.76 |
| K^+^ (μg·g^-1^) | 0.34 | 0.03* |  | 0.37 | 0.03* |
| Ca^2+^ (μg·g^-1^) | -0.10 | 0.53 |  | 0.14 | 0.44 |
| V^4+^ (μg·g^-1^) | 0.29 | 0.06 |  | -0.10 | 0.58 |
| Cr^3+^ (μg·g^-1^) | 0.18 | 0.26 |  | 0.05 | 0.78 |
| Mn^2+^ (μg·g^-1^) | -0.19 | 0.22 |  | 0.55 | 0.00*** |
| Fe^2+^ (μg·g^-1^) | 0.02 | 0.90 |  | 0.10 | 0.56 |
| Co^2+^ (μg·g^-1^) | -0.19 | 0.23 |  | 0.02 | 0.92 |
| Ni^2+^ (μg·g^-1^) | -0.12 | 0.45 |  | -0.07 | 0.71 |
| Cu^2+^ (μg·g^-1^) | 0.03 | 0.84 |  | -0.22 | 0.22 |
| Zn^2+^ (μg·g^-1^) | 0.08 | 0.60 |  | 0.07 | 0.69 |
| As^3+^ (μg·g^-1^) | 0.25 | 0.11 |  | -0.06 | 0.76 |
| Se^2+^ (μg·g^-1^) | 0.24 | 0.13 |  | 0.16 | 0.38 |
| Mo^6+^ (μg·g^-1^) | 0.41 | 0.01** |  | -0.61 | 0.00*** |
| Ag^+^ (μg·g^-1^) | -0.18 | 0.25 |  | -0.26 | 0.15 |
| Cd^2+^ (μg·g^-1^) | 0.25 | 0.11 |  | 0.12 | 0.51 |
| Sb^3+^ (μg·g^-1^) | -0.01 | 0.97 |  | -0.51 | 0.00** |
| Ba^2+^ (μg·g^-1^) | -0.23 | 0.15 |  | -0.25 | 0.17 |
| Tl^+^ (μg·g^-1^) | 0.40 | 0.01** |  | -0.04 | 0.83 |
| Pb^2+^ (μg·g^-1^) | -0.02 | 0.91 |  | -0.09 | 0.60 |
| Th^4+^ (μg·g^-1^) | -0.16 | 0.32 |  | -0.26 | 0.15 |
| U^3+^ (μg·g^-1^) | -0.40 | 0.01** |  | -0.34 | 0.05 |
| Sc^3+^ (μg·g^-1^) | 0.05 | 0.74 |  | -0.15 | 0.40 |
| Y^3+^ (μg·g^-1^) | 0.03 | 0.85 |  | -0.01 | 0.95 |
| La^3+^ (μg·g^-1^) | -0.10 | 0.52 |  | -0.04 | 0.83 |
| Ce^3+^ (μg·g^-1^) | -0.15 | 0.34 |  | -0.10 | 0.58 |
| Pr^3+^ (μg·g^-1^) | -0.06 | 0.69 |  | 0.00 | 1.00 |
| Nd^3+^ (μg·g^-1^) | -0.05 | 0.73 |  | 0.02 | 0.93 |
| Sm^3+^ (μg·g^-1^) | -0.03 | 0.86 |  | 0.02 | 0.91 |
| Eu^2+^ (μg·g^-1^) | 0.01 | 0.95 |  | 0.02 | 0.93 |
| Gd^3+^ (μg·g^-1^) | -0.06 | 0.70 |  | -0.01 | 0.94 |
| Tb^3+^ (μg·g^-1^) | 0.06 | 0.72 |  | 0.02 | 0.89 |
| Dy^3+^ (μg·g^-1^) | 0.09 | 0.57 |  | 0.05 | 0.77 |
| Ho^3+^ (μg·g^-1^) | 0.11 | 0.49 |  | 0.05 | 0.77 |
| Er^3+^ (μg·g^-1^) | 0.12 | 0.46 |  | 0.05 | 0.78 |
| Tm^3+^ (μg·g^-1^) | 0.12 | 0.44 |  | 0.00 | 0.99 |
| Yb^2+^ (μg·g^-1^) | 0.13 | 0.42 |  | 0.05 | 0.79 |
| Lu^3+^ (μg·g^-1^) | 0.17 | 0.29 |  | 0.03 | 0.87 |

*, *p* < 0.05; **, *p* < 0.01; ***, *p* < 0.001.

**Table S2** Linear correlation analysis between environmental variables and the relative abundances of dominant microbial phyla in the seepage area (ROV5) and non-seepage area (ROV3). ACC refers to archaeal community composition, BCC to bacterial community composition, and ECC to eukaryotic community composition.

| Environmental factors | ACC | | | | |  | BCC | | | | |  | ECC | | | | |
| --- | --- | --- | --- | --- | --- | --- | --- | --- | --- | --- | --- | --- | --- | --- | --- | --- | --- |
|  | Crenarchaeota | Asgardarchaeota | Nanoarchaeota | Thermoplasmatota | Halobacterota |  | Gammaproteobacteria | Chloroflexi | Caldatribacteriota | Desulfobacterota | Acidobacteriota |  | Ascomycota | Protalveolata | Cercozoa | Dinoflagellata | Retaria |
| depth (m) | -0.22 | 0.36** | 0.21 | -0.44*** | 0.02 |  | 0.53*** | -0.29* | -0.10 | -0.08 | -0.51*** |  | 0.42*** | -0.52*** | 0.62*** | 0.28* | 0.41*** |
| SO_4_^2^ (μmol·L^-1^) | 0.02 | -0.19 | -0.02 | 0.21 | -0.01 |  | -0.26* | 0.10 | 0.12 | 0.06 | 0.20 |  | -0.31** | 0.08 | -0.07 | -0.09 | -0.14 |
| Si (μmol·L^-1^) | 0.14 | -0.09 | -0.08 | -0.13 | -0.04 |  | 0.02 | -0.07 | 0.07 | -0.15 | 0.06 |  | -0.18 | -0.04 | 0.01 | 0.07 | 0.02 |
| SRP (μmol·L^-1^) | -0.10 | -0.08 | 0.05 | 0.01 | 0.18 |  | 0.16 | -0.14 | -0.03 | 0.01 | -0.17 |  | 0.06 | -0.10 | 0.02 | -0.12 | 0.01 |
| NH_4_^+^-N (μmol·L^-1^) | 0.20 | 0.08 | -0.17 | -0.07 | -0.27* |  | 0.37** | 0.03 | -0.31** | -0.32** | -0.09 |  | 0.14 | -0.16 | 0.36** | 0.33** | 0.06 |
| TN (%) | 0.13 | -0.35** | -0.15 | 0.22 | 0.04 |  | -0.35** | 0.16 | 0.11 | -0.01 | 0.38*** |  | -0.46*** | 0.27* | -0.09 | -0.06 | -0.16 |
| TC (%) | 0.09 | -0.44*** | -0.03 | 0.07 | 0.09 |  | -0.23* | 0.10 | 0.02 | 0.03 | 0.28* |  | -0.42*** | 0.27* | 0.07 | -0.05 | 0.04 |
| TOC (%) | 0.09 | -0.21 | -0.14 | 0.20 | 0.03 |  | -0.20 | 0.08 | 0.18 | 0.04 | 0.17 |  | -0.14 | -0.18 | -0.12 | 0.20 | -0.08 |
| TIC (%) | 0.06 | -0.41*** | 0.02 | 0.00 | 0.09 |  | -0.17 | 0.08 | -0.06 | 0.01 | 0.24* |  | -0.42*** | 0.39*** | 0.13 | -0.15 | 0.08 |
| C/N | -0.02 | -0.32** | 0.11 | -0.06 | 0.11 |  | -0.09 | 0.03 | -0.01 | 0.09 | 0.10 |  | -0.24* | 0.14 | 0.17 | -0.03 | 0.20 |
| pH | 0.27* | 0.30* | -0.21 | -0.05 | -0.53*** |  | 0.44*** | 0.19 | -0.60*** | -0.40*** | -0.07 |  | 0.21 | -0.10 | 0.38*** | 0.19 | 0.04 |
| Conductivity (mS·cm^-1^) | 0.30** | -0.09 | -0.38*** | 0.28* | -0.25* |  | -0.06 | 0.26* | -0.24* | -0.18 | 0.24* |  | -0.23* | 0.11 | 0.03 | 0.18 | -0.08 |
| Mg^2+^ (μg·g^-1^) | -0.10 | 0.08 | 0.23* | -0.31** | -0.13 |  | 0.23* | -0.06 | -0.18 | -0.02 | -0.18 |  | 0.08 | -0.15 | 0.29* | 0.00 | 0.18 |
| Al^3+^ (μg·g^-1^) | 0.19 | 0.05 | -0.20 | 0.07 | -0.22 |  | -0.01 | 0.29* | -0.22 | -0.14 | 0.06 |  | -0.05 | 0.02 | 0.04 | -0.17 | -0.08 |
| K^+^ (μg·g^-1^) | -0.32** | 0.16 | 0.46*** | -0.29* | 0.10 |  | 0.05 | -0.19 | 0.08 | 0.23* | -0.17 |  | 0.22 | -0.16 | 0.08 | -0.13 | 0.15 |
| Ca^2+^ (μg·g^-1^) | 0.07 | -0.43*** | 0.02 | -0.01 | 0.03 |  | -0.17 | 0.11 | -0.05 | 0.04 | 0.23* |  | -0.43*** | 0.22 | 0.13 | -0.12 | 0.04 |
| V^4+^ (μg·g^-1^) | -0.09 | 0.19 | -0.06 | 0.00 | 0.07 |  | -0.05 | 0.12 | 0.06 | 0.07 | -0.11 |  | 0.07 | -0.12 | 0.15 | 0.00 | -0.07 |
| Cr^3+^ (μg·g^-1^) | -0.19 | 0.27* | 0.17 | -0.13 | -0.02 |  | 0.05 | -0.03 | -0.03 | 0.13 | -0.18 |  | 0.16 | -0.11 | -0.04 | -0.08 | 0.01 |
| Mn^2+^ (μg·g^-1^) | 0.25* | -0.24* | 0.04 | -0.27* | -0.01 |  | -0.05 | -0.20 | -0.11 | 0.02 | -0.06 |  | -0.14 | 0.42*** | -0.02 | -0.10 | 0.10 |
| Fe^2+^ (μg·g^-1^) | -0.19 | 0.14 | 0.20 | -0.11 | 0.04 |  | -0.04 | -0.02 | 0.04 | 0.19 | -0.14 |  | 0.14 | -0.06 | -0.09 | -0.11 | -0.02 |
| Co^2+^ (μg·g^-1^) | 0.01 | -0.09 | 0.12 | -0.15 | 0.00 |  | -0.04 | -0.08 | -0.07 | 0.13 | -0.06 |  | -0.06 | 0.20 | -0.11 | -0.16 | -0.01 |
| Ni^2+^ (μg·g^-1^) | -0.04 | -0.31** | 0.06 | -0.05 | 0.21 |  | -0.16 | -0.12 | 0.18 | 0.16 | 0.05 |  | -0.30** | 0.14 | 0.04 | -0.11 | 0.03 |
| Cu^2+^ (μg·g^-1^) | -0.02 | -0.14 | -0.11 | 0.17 | 0.11 |  | -0.19 | 0.10 | 0.23* | 0.05 | 0.10 |  | -0.15 | -0.12 | 0.00 | -0.01 | -0.04 |
| Zn^2+^ (μg·g^-1^) | -0.14 | -0.10 | 0.14 | -0.11 | 0.12 |  | -0.10 | -0.01 | 0.13 | 0.12 | 0.00 |  | -0.17 | -0.03 | 0.05 | -0.14 | 0.04 |
| As^3+^ (μg·g^-1^) | -0.11 | 0.32** | 0.01 | 0.01 | 0.03 |  | 0.12 | -0.11 | 0.14 | -0.01 | -0.28* |  | 0.31** | -0.24* | 0.03 | 0.13 | -0.06 |
| Se^2+^ (μg·g^-1^) | -0.03 | -0.01 | -0.04 | 0.05 | -0.05 |  | -0.02 | 0.16 | 0.04 | -0.01 | 0.01 |  | -0.05 | -0.21 | 0.17 | 0.02 | 0.12 |
| Mo^6+^ (μg·g^-1^) | -0.16 | 0.07 | 0.04 | 0.17 | 0.13 |  | -0.26* | -0.02 | 0.37*** | 0.12 | 0.06 |  | -0.16 | 0.05 | 0.07 | -0.06 | -0.10 |
| Ag^+^ (μg·g^-1^) | 0.21 | -0.32** | -0.21 | 0.10 | -0.05 |  | -0.23* | 0.28* | -0.07 | -0.02 | 0.36** |  | -0.45*** | 0.35** | 0.00 | -0.10 | -0.12 |
| Cd^2+^ (μg·g^-1^) | -0.02 | -0.04 | -0.12 | 0.02 | 0.07 |  | 0.01 | 0.16 | 0.06 | -0.08 | -0.09 |  | -0.17 | -0.12 | 0.22 | 0.10 | -0.01 |
| Sb^3+^ (μg·g^-1^) | 0.25* | -0.28* | -0.30** | 0.25* | -0.08 |  | -0.24* | 0.26* | 0.07 | -0.16 | 0.26* |  | -0.26* | 0.10 | 0.08 | 0.09 | -0.17 |
| Ba^2+^ (μg·g^-1^) | 0.30** | -0.17 | -0.29* | 0.16 | -0.16 |  | -0.05 | 0.14 | -0.21 | -0.18 | 0.20 |  | -0.31** | 0.49*** | 0.03 | -0.13 | -0.10 |
| Tl^+^ (μg·g^-1^) | -0.20 | 0.03 | 0.03 | -0.01 | 0.24* |  | -0.04 | -0.11 | 0.33** | 0.09 | -0.10 |  | -0.09 | -0.27* | 0.28* | 0.08 | 0.08 |
| Pb^2+^ (μg·g^-1^) | -0.04 | 0.18 | -0.02 | -0.02 | -0.05 |  | -0.04 | 0.09 | 0.06 | 0.00 | -0.05 |  | 0.05 | -0.14 | -0.12 | -0.11 | -0.09 |
| Th^4+^ (μg·g^-1^) | -0.10 | 0.06 | 0.07 | 0.00 | 0.07 |  | -0.19 | 0.06 | 0.13 | 0.17 | 0.03 |  | -0.09 | 0.10 | -0.17 | -0.24* | -0.10 |
| U^3+^ (μg·g^-1^) | 0.24* | -0.22 | -0.29* | 0.42*** | -0.21 |  | -0.26* | 0.40** | 0.03 | -0.14 | 0.39*** |  | -0.40*** | 0.05 | -0.16 | -0.17 | -0.29* |
| Sc^3+^ (μg·g^-1^) | -0.12 | 0.22 | 0.05 | -0.05 | 0.01 |  | -0.03 | 0.03 | -0.01 | 0.10 | -0.07 |  | 0.07 | -0.02 | -0.09 | -0.11 | -0.07 |
| Y^3+^ (μg·g^-1^) | -0.11 | 0.09 | 0.09 | -0.03 | -0.02 |  | -0.06 | 0.06 | -0.02 | 0.14 | -0.07 |  | 0.02 | -0.03 | -0.08 | -0.14 | -0.05 |
| La^3+^ (μg·g^-1^) | 0.06 | 0.24* | -0.04 | 0.04 | -0.23* |  | -0.02 | 0.21 | -0.17 | -0.02 | -0.03 |  | 0.11 | -0.04 | -0.13 | -0.13 | -0.13 |
| Ce^3+^ (μg·g^-1^) | 0.06 | 0.25* | -0.06 | 0.08 | -0.24* |  | -0.03 | 0.21 | -0.13 | -0.04 | -0.02 |  | 0.11 | -0.06 | -0.18 | -0.13 | -0.17 |
| Pr^3+^ (μg·g^-1^) | 0.03 | 0.26* | -0.01 | 0.01 | -0.22 |  | 0.01 | 0.18 | -0.16 | -0.02 | -0.06 |  | 0.14 | -0.08 | -0.13 | -0.12 | -0.12 |
| Nd^3+^ (μg·g^-1^) | 0.03 | 0.26* | -0.01 | 0.00 | -0.22 |  | 0.02 | 0.17 | -0.16 | -0.02 | -0.08 |  | 0.15 | -0.09 | -0.12 | -0.13 | -0.11 |
| Sm^3+^ (μg·g^-1^) | 0.02 | 0.25* | -0.01 | -0.01 | -0.22 |  | 0.03 | 0.17 | -0.16 | -0.03 | -0.08 |  | 0.13 | -0.08 | -0.11 | -0.12 | -0.10 |
| Eu^2+^ (μg·g^-1^) | 0.02 | 0.18 | -0.01 | -0.02 | -0.20 |  | 0.02 | 0.17 | -0.17 | -0.04 | -0.04 |  | 0.07 | -0.03 | -0.07 | -0.12 | -0.09 |
| Gd^3+^ (μg·g^-1^) | 0.02 | 0.27* | -0.03 | 0.02 | -0.23* |  | 0.03 | 0.17 | -0.15 | -0.03 | -0.08 |  | 0.16 | -0.10 | -0.16 | -0.09 | -0.12 |
| Tb^3+^ (μg·g^-1^) | -0.05 | 0.25* | 0.03 | -0.03 | -0.16 |  | 0.04 | 0.11 | -0.11 | 0.02 | -0.12 |  | 0.15 | -0.12 | -0.10 | -0.09 | -0.08 |
| Dy^3+^ (μg·g^-1^) | -0.06 | 0.24* | 0.05 | -0.05 | -0.14 |  | 0.05 | 0.10 | -0.09 | 0.02 | -0.13 |  | 0.16 | -0.13 | -0.09 | -0.09 | -0.06 |
| Ho^3+^ (μg·g^-1^) | -0.09 | 0.21 | 0.07 | -0.06 | -0.10 |  | 0.03 | 0.07 | -0.07 | 0.06 | -0.14 |  | 0.13 | -0.12 | -0.07 | -0.09 | -0.05 |
| Er^3+^ (μg·g^-1^) | -0.08 | 0.22 | 0.06 | -0.05 | -0.12 |  | 0.04 | 0.08 | -0.08 | 0.05 | -0.14 |  | 0.14 | -0.13 | -0.07 | -0.09 | -0.06 |
| Tm^3+^ (μg·g^-1^) | -0.06 | 0.20 | 0.04 | -0.04 | -0.11 |  | 0.01 | 0.09 | -0.08 | 0.07 | -0.12 |  | 0.12 | -0.08 | -0.06 | -0.09 | -0.08 |
| Yb^2+^ (μg·g^-1^) | -0.07 | 0.20 | 0.06 | -0.05 | -0.11 |  | 0.02 | 0.08 | -0.06 | 0.06 | -0.13 |  | 0.11 | -0.13 | -0.06 | -0.08 | -0.06 |
| Lu^3+^ (μg·g^-1^) | -0.05 | 0.18 | 0.04 | -0.06 | -0.13 |  | 0.04 | 0.09 | -0.09 | 0.03 | -0.11 |  | 0.10 | -0.09 | -0.04 | -0.06 | -0.07 |

*, *p* < 0.05; **, *p* < 0.01; ***, *p* < 0.001.

**Table S3** Significance tests of microbial community differences between the seepage area (ROV5) and the non-seepage area (ROV3). Groups with significant differences (*p* < 0.05) are highlighted. ACC represents archaeal community composition, BCC represents bacterial community composition, PCC represents prokaryotic community composition, and ECC represents eukaryotic community composition.

| Index | MRPP | |  | ANOSIM | |  | PERMANOVA | |
| --- | --- | --- | --- | --- | --- | --- | --- | --- |
|  | r | p |  | r | p |  | r | p |
| PCC | 0.0797 | 0.001*** |  | 0.4372 | 0.001*** |  | 0.3953 | 0.001*** |
| ECC | -0.0005 | 0.532 |  | 0.013 | 0.226 |  | 0.1127 | 0.545 |
| ACC | 0.0891 | 0.001*** |  | 0.618 | 0.001*** |  | 0.4229 | 0.001*** |
| BCC | 0.0785 | 0.001*** |  | 0.404 | 0.001*** |  | 0.3908 | 0.001*** |

*, *p* < 0.05; **, *p* < 0.01; ***, *p* < 0.001.

**Table S4** Partial Mantel tests based on Spearman's correlations (r values) between transformed environmental variables and microbial community composition in the non-seepage area (ROV3).

| Environmental factors | PCC | |  | ACC | |  | BCC | |
| --- | --- | --- | --- | --- | --- | --- | --- | --- |
|  | r | p |  | r | p |  | r | p |
| Depth (m) | 0.469 | 0.001 *** |  | 0.589 | 0.001 *** |  | 0.444 | 0.001 *** |
| SO_4_^2-^ (μmol·L^-1^) | 0.098 | 0.133 |  | 0.148 | 0.066 |  | 0.096 | 0.139 |
| Si (μmol·L^-1^) | -0.028 | 0.679 |  | -0.012 | 0.570 |  | -0.027 | 0.670 |
| SRP (μmol·L^-1^) | 0.095 | 0.142 |  | 0.212 | 0.024* |  | 0.088 | 0.160 |
| NH_4_^+^-N (μmol·L^-1^) | -0.044 | 0.644 |  | -0.087 | 0.801 |  | -0.034 | 0.586 |
| TN (%) | -0.113 | 0.907 |  | 0.001 | 0.476 |  | -0.117 | 0.917 |
| TOC (%) | -0.122 | 0.901 |  | -0.099 | 0.809 |  | -0.120 | 0.899 |
| pH | 0.309 | 0.001*** |  | 0.421 | 0.001*** |  | 0.290 | 0.002** |
| Conductivity (mS·cm^-1^) | -0.286 | 1.000 |  | -0.229 | 0.998 |  | -0.279 | 1.000 |
| Mg^2+^ (μg·g^-1^) | -0.093 | 0.821 |  | 0.157 | 0.058 |  | -0.110 | 0.884 |
| Al^3+^ (μg·g^-1^) | -0.106 | 0.943 |  | -0.060 | 0.790 |  | -0.106 | 0.950 |
| K^+^ (μg·g^-1^) | -0.166 | 0.977 |  | -0.144 | 0.936 |  | -0.163 | 0.978 |
| V^4+^ (μg·g^-1^) | -0.029 | 0.648 |  | -0.163 | 0.988 |  | -0.021 | 0.596 |
| Mn^2+^ (μg·g^-1^) | 0.462 | 0.018* |  | 0.525 | 0.014* |  | 0.445 | 0.017* |
| Fe^2+^ (μg·g^-1^) | -0.106 | 0.883 |  | -0.066 | 0.752 |  | -0.106 | 0.884 |
| Co^2+^ (μg·g^-1^) | 0.123 | 0.073* |  | 0.158 | 0.037* |  | 0.117 | 0.086 |
| Ni^2+^ (μg·g^-1^) | -0.186 | 0.989 |  | -0.210 | 0.986 |  | -0.184 | 0.986 |
| Cu^2+^ (μg·g^-1^) | -0.242 | 1.000 |  | -0.227 | 0.998 |  | -0.235 | 1.000 |
| Zn^2+^ (μg·g^-1^) | -0.341 | 1.000 |  | -0.389 | 1.000 |  | -0.331 | 1.000 |
| As^3+^ (μg·g^-1^) | 0.029 | 0.352 |  | -0.007 | 0.522 |  | 0.034 | 0.336 |
| Se^3+^ (μg·g^-1^) | 0.009 | 0.425 |  | -0.078 | 0.863 |  | 0.016 | 0.388 |
| Mo^6+^ (μg·g^-1^) | 0.111 | 0.154 |  | 0.196 | 0.069 |  | 0.094 | 0.178 |
| Ag^+^ (μg·g^-1^) | -0.115 | 0.923 |  | -0.151 | 0.965 |  | -0.110 | 0.904 |
| Cd^2+^ (μg·g^-1^) | 0.101 | 0.126 |  | -0.005 | 0.461 |  | 0.106 | 0.125 |
| Sb^3+^ (μg·g^-1^) | -0.201 | 0.999 |  | -0.205 | 0.994 |  | -0.194 | 0.999 |
| Tl^+^ (μg·g^-1^) | -0.079 | 0.856 |  | -0.094 | 0.891 |  | -0.076 | 0.847 |
| U^3+^ (μg·g^-1^) | 0.199 | 0.032* |  | 0.184 | 0.051 |  | 0.194 | 0.033* |

*, *p* < 0.05; **, *p* < 0.01; ***, *p* < 0.001.

**Table S5** Partial Mantel tests identified Spearman's correlations (r values) between transformed environmental variables and microbial community composition in the seepage area (ROV5).

| Environmental factors | PCC | |  | ACC | |  | BCC | |
| --- | --- | --- | --- | --- | --- | --- | --- | --- |
|  | r | p |  | r | p |  | r | p |
| Depth (m) | 0.664 | 0.001*** |  | 0.620 | 0.001*** |  | 0.633 | 0.001*** |
| SO_4_^2-^ (μmol·L^-1^) | 0.020 | 0.385 |  | 0.040 | 0.328 |  | 0.022 | 0.374 |
| Si (μmol·L^-1^) | 0.003 | 0.456 |  | -0.036 | 0.741 |  | 0.010 | 0.399 |
| SRP (μmol·L^-1^) | -0.130 | 0.969 |  | -0.106 | 0.824 |  | -0.126 | 0.938 |
| NH_4_^+^-N (μmol·L^-1^) | 0.269 | 0.001*** |  | 0.283 | 0.001*** |  | 0.245 | 0.004** |
| TN (%) | 0.175 | 0.029* |  | 0.139 | 0.079 |  | 0.173 | 0.031* |
| TOC (%) | 0.234 | 0.010* |  | 0.174 | 0.039* |  | 0.234 | 0.011* |
| C/N (%) | 0.086 | 0.137 |  | 0.102 | 0.111 |  | 0.084 | 0.145 |
| pH | 0.164 | 0.042* |  | 0.069 | 0.240 |  | 0.180 | 0.033* |
| Conductivity  (mS·cm^-1^) | -0.022 | 0.565 |  | 0.057 | 0.276 |  | -0.022 | 0.561 |
| Mg^2+^ (μg·g^-1^) | -0.051 | 0.716 |  | -0.051 | 0.691 |  | -0.049 | 0.703 |
| K^+^ (μg·g^-1^) | -0.051 | 0.692 |  | 0.016 | 0.404 |  | -0.060 | 0.725 |
| Ca^2+^ (μg·g^-1^) | -0.043 | 0.704 |  | -0.053 | 0.745 |  | -0.036 | 0.670 |
| Mn^2+^ (μg·g^-1^) | 0.007 | 0.471 |  | 0.005 | 0.473 |  | 0.005 | 0.489 |
| Zn^2+^ (μg·g^-1^) | -0.317 | 1.000 |  | -0.218 | 0.998 |  | -0.321 | 1.000 |
| As^3+^ (μg·g^-1^) | 0.034 | 0.348 |  | 0.029 | 0.301 |  | 0.034 | 0.340 |
| Se^2+^ (μg·g^-1^) | -0.190 | 0.998 |  | -0.218 | 0.999 |  | -0.179 | 0.994 |
| Mo^6+^ (μg·g^-1^) | 0.261 | 0.002** |  | 0.137 | 0.060 |  | 0.260 | 0.002** |
| Ag^+^ (μg·g^-1^) | 0.139 | 0.062 |  | 0.048 | 0.286 |  | 0.143 | 0.061 |
| Cd^2+^ (μg·g^-1^) | -0.168 | 0.987 |  | -0.145 | 0.967 |  | -0.163 | 0.983 |
| Sb^3+^ (μg·g^-1^) | 0.057 | 0.248 |  | 0.050 | 0.267 |  | 0.056 | 0.247 |
| Ba^2+^ (μg·g^-1^) | 0.060 | 0.233 |  | -0.003 | 0.455 |  | 0.065 | 0.226 |
| Tl^+^ (μg·g^-1^) | -0.112 | 0.936 |  | -0.076 | 0.812 |  | -0.110 | 0.928 |
| U^3+^ (μg·g^-1^) | -0.013 | 0.505 |  | 0.026 | 0.328 |  | -0.022 | 0.532 |

*, *p* < 0.05; **, *p* < 0.01; ***, *p* < 0.001.

**Table S6** Specialized microbial taxa (ASVs) in seepage (ROV5) and non-seepage (ROV3) areas.

| ROV3 |  | Kingdom | Phylum | Class | | Order | | Family | Genus |
| --- | --- | --- | --- | --- | --- | --- | --- | --- | --- |
|  | ASV11 | Archaea | Crenarchaeota | Bathyarchaeia | Bathyarchaeia | | Bathyarchaeia | | Bathyarchaeia |
|  | ASV116 | Archaea | Crenarchaeota | Bathyarchaeia | Bathyarchaeia | | Bathyarchaeia | | Bathyarchaeia |
|  | ASV150 | Archaea | Crenarchaeota | Bathyarchaeia | Bathyarchaeia | | Bathyarchaeia | | Bathyarchaeia |
|  | ASV221 | Archaea | Hadarchaeota | Hadarchaeia | Hadarchaeia | | Hadarchaeales | | Hadarchaeales |
|  | ASV49 | Archaea | Hadarchaeota | Hadarchaeia | Hadarchaeia | | Hadarchaeales | | Hadarchaeales |
|  | ASV59 | Archaea | Nanoarchaeota | Nanoarchaeia | Woesearchaeales | | SCGC_AAA011-D5 | | SCGC_AAA011-D5 |
|  | ASV704 | Archaea | Archaea_unclassified | Archaea_unclassified | Archaea_unclassified | | Archaea_unclassified | | Archaea_unclassified |
|  | ASV72 | Archaea | Crenarchaeota | Bathyarchaeia | Bathyarchaeia | | Bathyarchaeia | | Bathyarchaeia |
|  | ASV846 | Archaea | Crenarchaeota | Bathyarchaeia | Bathyarchaeia | | Bathyarchaeia | | Bathyarchaeia |
|  | ASV102 | Bacteria | Planctomycetota | Phycisphaerae | MSBL9 | | SG8-4 | | SG8-4 |
|  | ASV103 | Bacteria | Chloroflexi | Dehalococcoidia | DscP2 | | DscP2 | | DscP2 |
|  | ASV12 | Bacteria | Aerophobota | Aerophobia | Aerophobales | | Aerophobales | | Aerophobales |
|  | ASV122 | Bacteria | Chloroflexi | Dehalococcoidia | FW22 | | FW22 | | FW22 |
|  | ASV123 | Bacteria | Chloroflexi | Dehalococcoidia | GIF3 | | GIF3 | | GIF3 |
|  | ASV136 | Bacteria | Chloroflexi | Dehalococcoidia | GIF9 | | GIF9 | | GIF9 |
|  | ASV148 | Bacteria | Actinobacteriota | WCHB1-81 | WCHB1-81 | | WCHB1-81 | | WCHB1-81 |
|  | ASV154 | Bacteria | Bacteria_unclassified | Bacteria_unclassified | Bacteria_unclassified | | Bacteria_unclassified | | Bacteria_unclassified |
|  | ASV158 | Bacteria | Chloroflexi | Anaerolineae | MSB-5B2 | | MSB-5B2 | | MSB-5B2 |
|  | ASV162 | Bacteria | Desulfobacterota | Desulfobacteria | Desulfatiglandales | | Desulfatiglandaceae | | Desulfatiglans |
|  | ASV165 | Bacteria | Desulfobacterota | Desulfobacteria | Desulfobacterales | | Desulfosarcinaceae | | SEEP-SRB1 |
|  | ASV170 | Bacteria | Chloroflexi | Dehalococcoidia | Napoli-4B-65 | | Napoli-4B-65 | | Napoli-4B-65 |
|  | ASV179 | Bacteria | Chloroflexi | Dehalococcoidia | MSBL5 | | MSBL5 | | MSBL5 |
|  | ASV203 | Bacteria | Patescibacteria | Parcubacteria | Parcubacteria_unclassified | | Parcubacteria_unclassified | | Parcubacteria_unclassified |
|  | ASV215 | Bacteria | Chloroflexi | Dehalococcoidia | GIF3 | | GIF3 | | GIF3 |
|  | ASV229 | Bacteria | Chloroflexi | Dehalococcoidia | Dehalococcoidia_unclassified | | Dehalococcoidia_unclassified | | Dehalococcoidia_unclassified |
|  | ASV236 | Bacteria | Chloroflexi | Dehalococcoidia | GIF3 | | GIF3 | | GIF3 |
|  | ASV24 | Bacteria | Chloroflexi | Dehalococcoidia | Dehalococcoidia_unclassified | | Dehalococcoidia_unclassified | | Dehalococcoidia_unclassified |
|  | ASV25 | Bacteria | Chloroflexi | Anaerolineae | Anaerolineae_unclassified | | Anaerolineae_unclassified | | Anaerolineae_unclassified |
|  | ASV251 | Bacteria | Chloroflexi | Dehalococcoidia | H3.93 | | H3.93 | | H3.93 |
|  | ASV265 | Bacteria | Chloroflexi | Anaerolineae | Anaerolineales | | Anaerolineaceae | | uncultured |
|  | ASV315 | Bacteria | Bacteria_unclassified | Bacteria_unclassified | Bacteria_unclassified | | Bacteria_unclassified | | Bacteria_unclassified |
|  | ASV32 | Bacteria | Chloroflexi | Anaerolineae | MSB-5B2 | | MSB-5B2 | | MSB-5B2 |
|  | ASV326 | Bacteria | Chloroflexi | Dehalococcoidia | Dehalococcoidia_unclassified | | Dehalococcoidia_unclassified | | Dehalococcoidia_unclassified |
|  | ASV362 | Bacteria | Proteobacteria | Alphaproteobacteria | Alphaproteobacteria_unclassified | | Alphaproteobacteria_unclassified | | Alphaproteobacteria_unclassified |
|  | ASV386 | Bacteria | Chloroflexi | Dehalococcoidia | Dehalococcoidia_unclassified | | Dehalococcoidia_unclassified | | Dehalococcoidia_unclassified |
|  | ASV398 | Bacteria | Chloroflexi | Dehalococcoidia | Dehalococcoidia_unclassified | | Dehalococcoidia_unclassified | | Dehalococcoidia_unclassified |
|  | ASV411 | Bacteria | Spirochaetota | Spirochaetia | Spirochaetales | | Spirochaetaceae | | uncultured |
|  | ASV415 | Bacteria | Chloroflexi | Dehalococcoidia | Sh765B-AG-111 | | Sh765B-AG-111 | | Sh765B-AG-111 |
|  | ASV42 | Bacteria | Chloroflexi | Dehalococcoidia | Napoli-4B-65 | | Napoli-4B-65 | | Napoli-4B-65 |
|  | ASV43 | Bacteria | Acidobacteriota | Aminicenantia | Aminicenantales | | Aminicenantales | | Aminicenantales |
|  | ASV432 | Bacteria | Spirochaetota | Spirochaetia | Spirochaetales | | Spirochaetaceae | | Spirochaeta |
|  | ASV46 | Bacteria | Elusimicrobiota | 45411 | 4-29 | | 4-29 | | 4-29 |
|  | ASV48 | Bacteria | Chloroflexi | Dehalococcoidia | GIF3 | | GIF3 | | GIF3 |
|  | ASV526 | Bacteria | Sva0485 | Sva0485_cl | Sva0485 | | Sva0485 | | Sva0485 |
|  | ASV53 | Bacteria | Bacteria_unclassified | Bacteria_unclassified | Bacteria_unclassified | | Bacteria_unclassified | | Bacteria_unclassified |
|  | ASV531 | Bacteria | Chloroflexi | Dehalococcoidia | Dehalococcoidia_unclassified | | Dehalococcoidia_unclassified | | Dehalococcoidia_unclassified |
|  | ASV536 | Bacteria | Chloroflexi | Dehalococcoidia | Dehalococcoidia_unclassified | | Dehalococcoidia_unclassified | | Dehalococcoidia_unclassified |
|  | ASV54 | Bacteria | Aerophobota | Aerophobia | Aerophobales | | Aerophobales | | Aerophobales |
|  | ASV546 | Bacteria | Chloroflexi | Dehalococcoidia | H3.93 | | H3.93 | | H3.93 |
|  | ASV60 | Bacteria | Acidobacteriota | Aminicenantia | Aminicenantales | | Aminicenantales | | Aminicenantales |
|  | ASV63 | Bacteria | Aerophobota | Aerophobia | Aerophobales | | Aerophobales | | Aerophobales |
|  | ASV64 | Bacteria | Chloroflexi | Dehalococcoidia | Dehalococcoidia_unclassified | | Dehalococcoidia_unclassified | | Dehalococcoidia_unclassified |
|  | ASV65 | Bacteria | Chloroflexi | Anaerolineae | SBR1031 | | SBR1031 | | SBR1031 |
|  | ASV68 | Bacteria | Chloroflexi | Dehalococcoidia | Sh765B-AG-111 | | Sh765B-AG-111 | | Sh765B-AG-111 |
|  | ASV7 | Bacteria | Chloroflexi | Dehalococcoidia | GIF3 | | GIF3 | | GIF3 |
|  | ASV71 | Bacteria | Aerophobota | Aerophobia | Aerophobales | | Aerophobales | | Aerophobales |
|  | ASV73 | Bacteria | Bacteria_unclassified | Bacteria_unclassified | Bacteria_unclassified | | Bacteria_unclassified | | Bacteria_unclassified |
|  | ASV74 | Bacteria | Desulfobacterota | Desulfobacteria | Desulfatiglandales | | Desulfatiglandaceae | | Desulfatiglans |
|  | ASV76 | Bacteria | Chloroflexi | Dehalococcoidia | H3.93 | | H3.93 | | H3.93 |
|  | ASV763 | Bacteria | Chloroflexi | Dehalococcoidia | Dehalococcoidia_unclassified | | Dehalococcoidia_unclassified | | Dehalococcoidia_unclassified |
|  | ASV79 | Bacteria | Chloroflexi | Dehalococcoidia | Sh765B-AG-111 | | Sh765B-AG-111 | | Sh765B-AG-111 |
|  | ASV81 | Bacteria | Chloroflexi | Dehalococcoidia | 1226B1H1-22-FL | | 1226B1H1-22-FL | | 1226B1H1-22-FL |
|  | ASV82 | Bacteria | Chloroflexi | Dehalococcoidia | Dehalococcoidia_unclassified | | Dehalococcoidia_unclassified | | Dehalococcoidia_unclassified |
|  | ASV84 | Bacteria | Bacteria_unclassified | Bacteria_unclassified | Bacteria_unclassified | | Bacteria_unclassified | | Bacteria_unclassified |
|  | ASV93 | Bacteria | Chloroflexi | Anaerolineae | MSB-5B2 | | MSB-5B2 | | MSB-5B2 |
|  | ASV96 | Bacteria | Acidobacteriota | Aminicenantia | Aminicenantales | | Aminicenantales | | Aminicenantales |
| ROV5 | ASV14 | Archaea | Nanoarchaeota | Nanoarchaeia | Woesearchaeales | | Woesearchaeales_unclassified | | Woesearchaeales_unclassified |
|  | ASV16 | Archaea | Asgardarchaeota | Lokiarchaeia | Lokiarchaeia | | Lokiarchaeia | | Lokiarchaeia |
|  | ASV20 | Archaea | Halobacterota | Methanosarcinia | Methanosarciniales | | Methanosarcinaceae | | ANME-3 |
|  | ASV264 | Archaea | Asgardarchaeota | Lokiarchaeia | Lokiarchaeia | | Lokiarchaeia | | Lokiarchaeia |
|  | ASV39 | Archaea | Halobacterota | Methanosarcinia | Methanosarciniales | | ANME-2a-2b | | ANME-2a-2b |
|  | ASV94 | Archaea | Thermoplasmatota | Thermoplasmata | Marine_Benthic_Group_D_and_DHVEG-1 | | Marine_Benthic_Group_D_and_DHVEG-1 | | Marine_Benthic_Group_D_and_DHVEG-1 |
|  | ASV10 | Bacteria | Desulfobacterota | Desulfobacteria | Desulfobacterales | | Desulfosarcinaceae | | SEEP-SRB1 |
|  | ASV108 | Bacteria | Bacteroidota | Bacteroidia | Bacteroidales | | Bacteroidales_unclassified | | Bacteroidales_unclassified |
|  | ASV109 | Bacteria | Acetothermia | Acetothermiia | Acetothermiia | | Acetothermiia | | Acetothermiia |
|  | ASV168 | Bacteria | Bacteria_unclassified | Bacteria_unclassified | Bacteria_unclassified | | Bacteria_unclassified | | Bacteria_unclassified |
|  | ASV175 | Bacteria | Chloroflexi | Dehalococcoidia | MSBL5 | | MSBL5 | | MSBL5 |
|  | ASV263 | Bacteria | Desulfobacterota | Desulfobacteria | Desulfobacterales | | uncultured | | uncultured |
|  | ASV28 | Bacteria | Patescibacteria | ABY1 | Candidatus_Falkowbacteria | | Candidatus_Falkowbacteria | | Candidatus_Falkowbacteria |
|  | ASV3 | Bacteria | Caldatribacteriota | JS1 | JS1 | | JS1 | | JS1 |
|  | ASV347 | Bacteria | Sva0485 | Sva0485_cl | Sva0485 | | Sva0485 | | Sva0485 |
|  | ASV37 | Bacteria | Desulfobacterota | Desulfobacteria | Desulfobacterales | | Desulfosarcinaceae | | SEEP-SRB1 |
|  | ASV374 | Bacteria | Spirochaetota | Spirochaetia | Spirochaetales | | Spirochaetaceae | | Sediminispirochaeta |
|  | ASV377 | Bacteria | Spirochaetota | Spirochaetia | Spirochaetales | | Spirochaetaceae | | Spirochaetaceae_unclassified |
|  | ASV44 | Bacteria | Actinobacteriota | WCHB1-81 | WCHB1-81 | | WCHB1-81 | | WCHB1-81 |
|  | ASV45 | Bacteria | Caldatribacteriota | JS1 | JS1 | | JS1 | | JS1 |
|  | ASV462 | Bacteria | Acetothermia | Acetothermiia | Acetothermiia | | Acetothermiia | | Acetothermiia |
|  | ASV47 | Bacteria | Chloroflexi | Anaerolineae | Anaerolineales | | Anaerolineaceae | | uncultured |
|  | ASV490 | Bacteria | Planctomycetota | Phycisphaerae | MSBL9 | | SG8-4 | | SG8-4 |
|  | ASV509 | Bacteria | Planctomycetota | Phycisphaerae | Phycisphaerales | | AKAU3564_sediment_group | | AKAU3564_sediment_group |
|  | ASV51 | Bacteria | Acidobacteriota | Aminicenantia | Aminicenantales | | Aminicenantales | | Aminicenantales |
|  | ASV790 | Bacteria | Spirochaetota | Spirochaetia | Spirochaetales | | Spirochaetaceae | | Spirochaeta |
|  | ASV9 | Bacteria | Desulfobacterota | Desulfobacteria | Desulfobacterales | | Desulfosarcinaceae | | SEEP-SRB1 |
|  | ASV90 | Bacteria | Desulfobacterota | Desulfobacteria | Desulfobacterales | | Desulfosarcinaceae | | uncultured |
|  | ASV97 | Bacteria | Desulfobacterota | Desulfobacteria | Desulfatiglandales | | Desulfatiglandaceae | | Desulfatiglans |


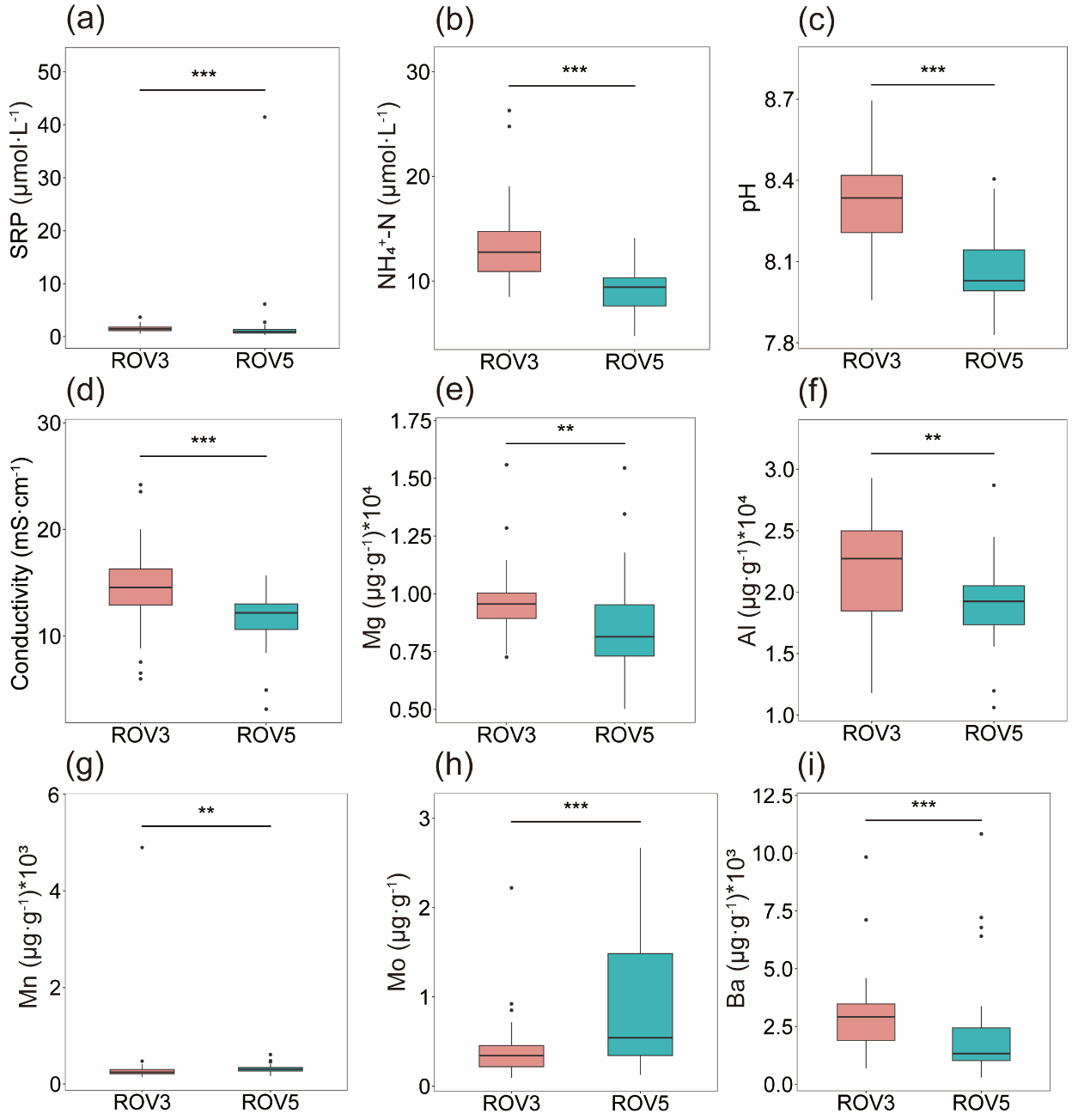


**Fig. S1** Comparison of environmental factors with significant differences between the seepage area (ROV5) and the non-seepage area (ROV3). Symbols indicate significance levels based on Wilcoxon tests. **, *p* < 0.01; ***, *p* < 0.001.


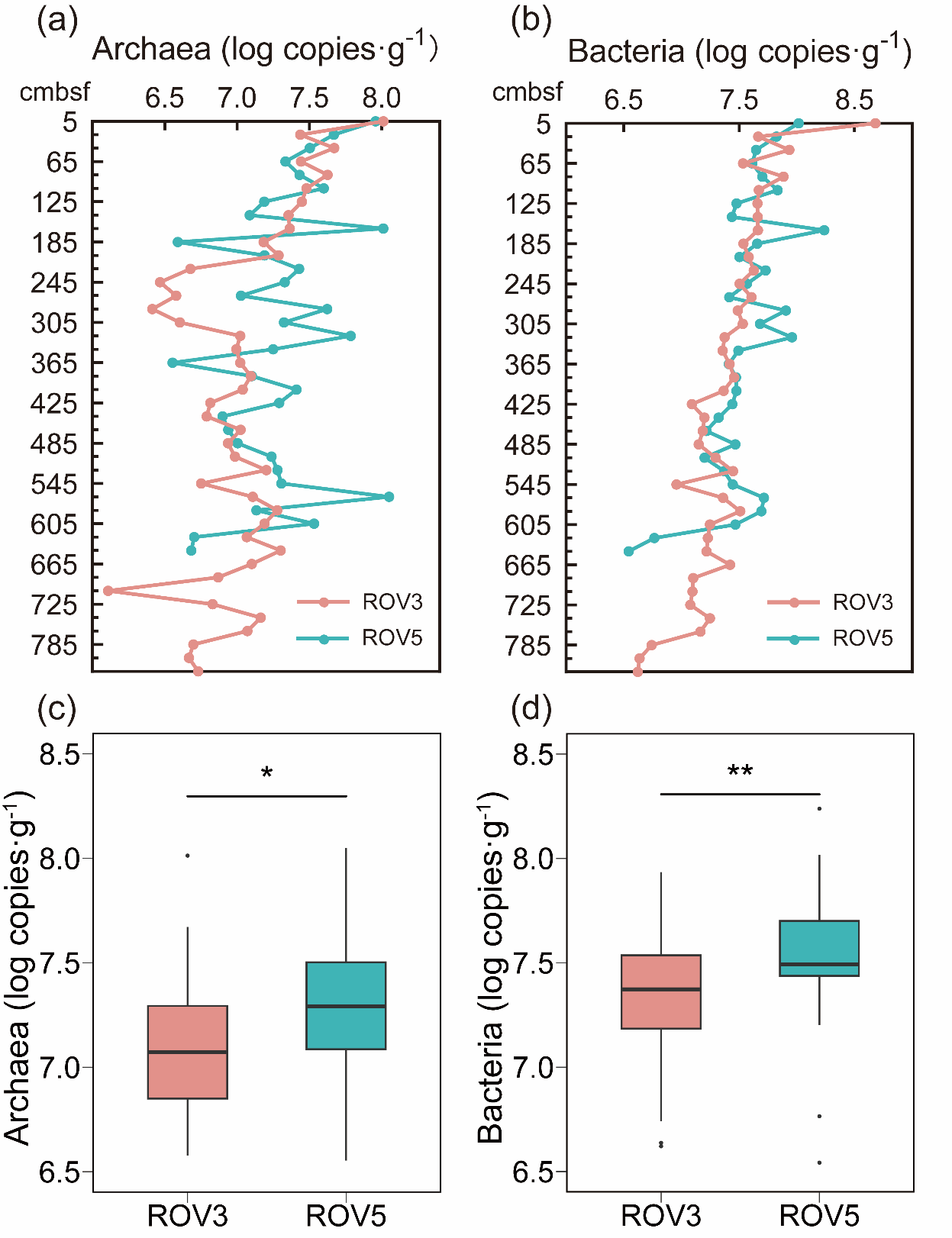


**Fig. S2** Archaeal and bacterial 16S rRNA gene abundances in the seepage area (ROV5) and non-seepage area (ROV3). Symbols indicate significance levels based on Wilcoxon tests. *, *p* < 0.05; **, *p* < 0.01.


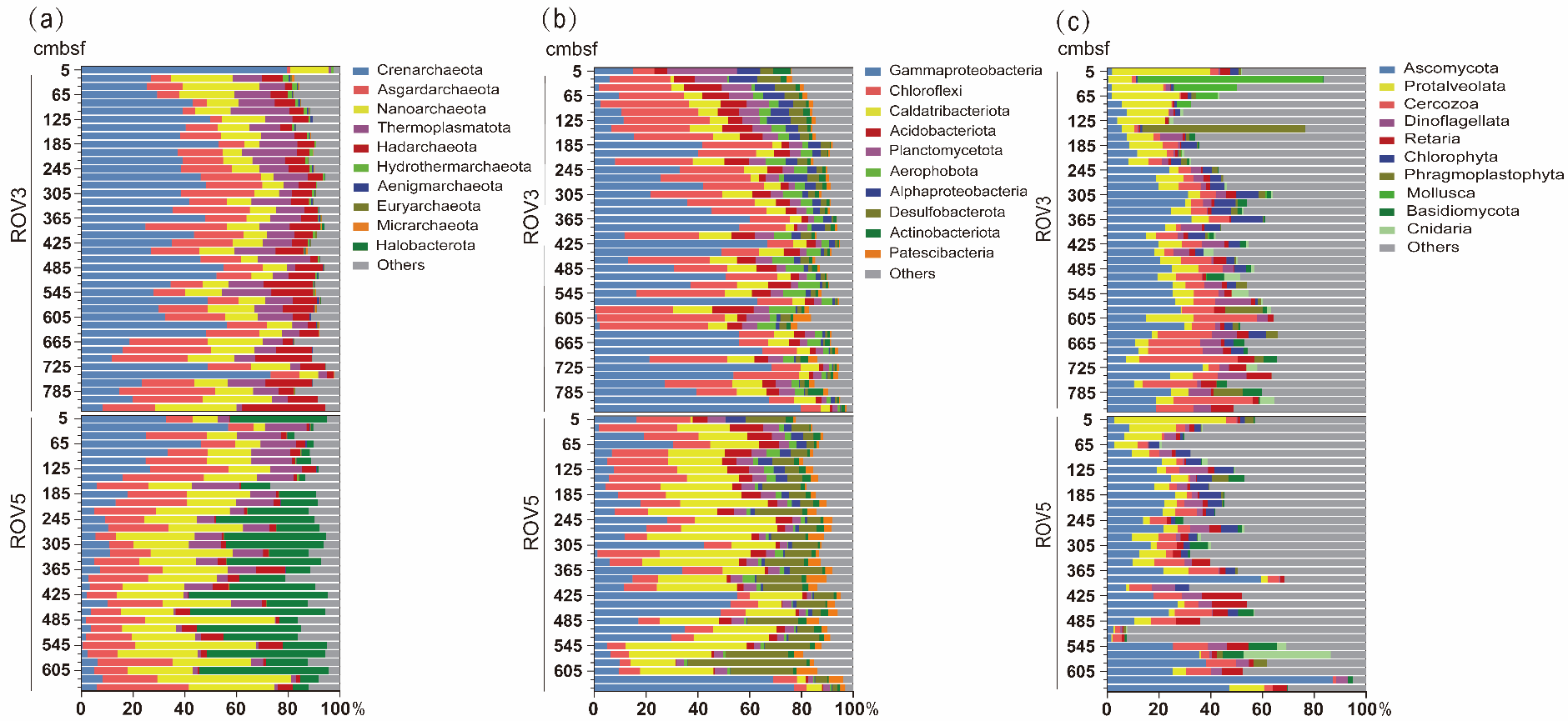


**Fig. S3** Relative abundances of microbial community compositions at the phylum or class level in the seepage area (ROV5) and non-seepage area (ROV3): (a) archaeal community composition, (b) bacterial community composition, and (c) eukaryotic community composition.


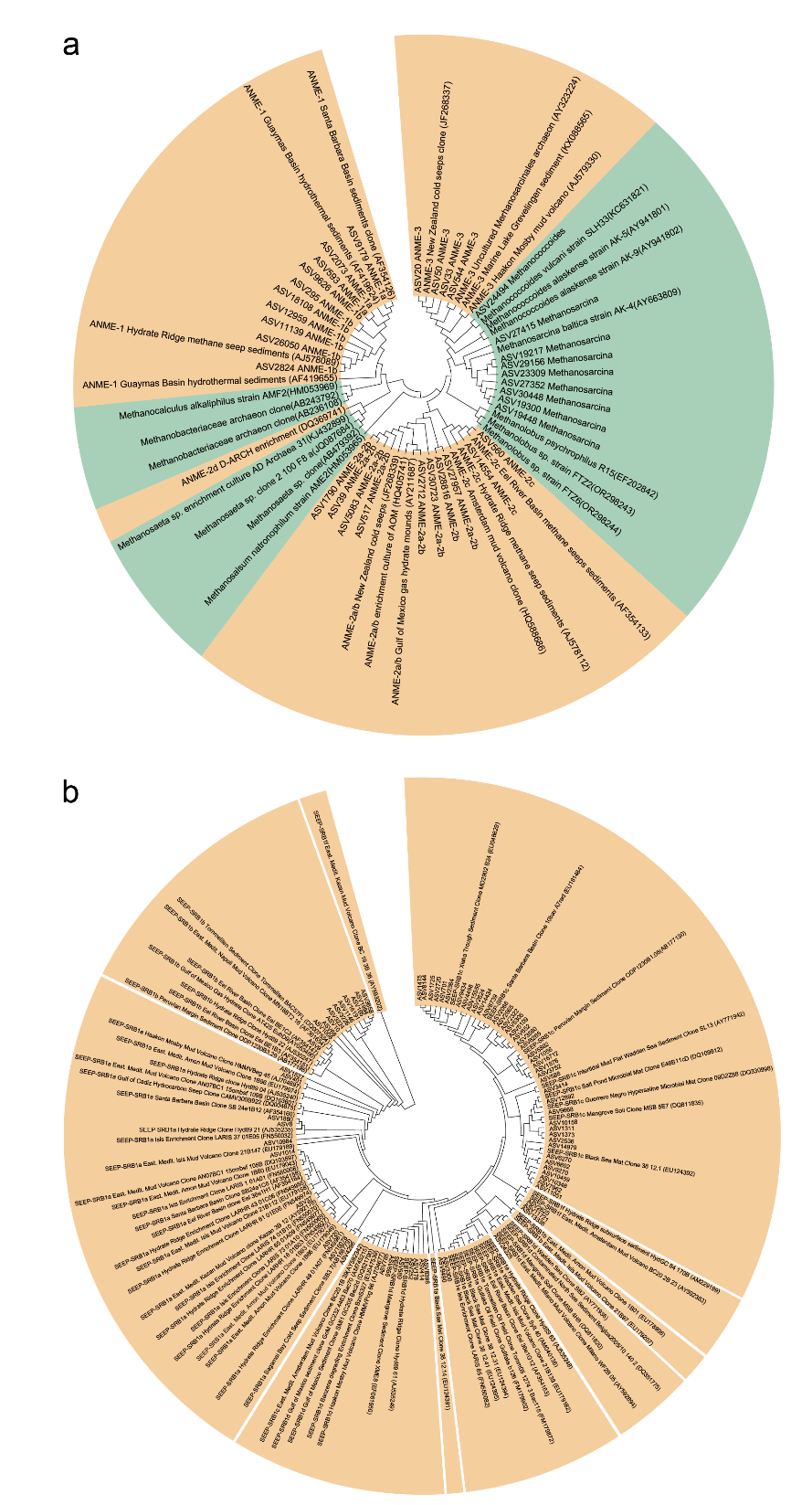


**Fig. S4** Neighbor-joining trees showing the phylogenetic relationships of ANME and methanogen (a), SRB1 and *Desulfobacteraceae* (b) 16S rRNA gene sequences.


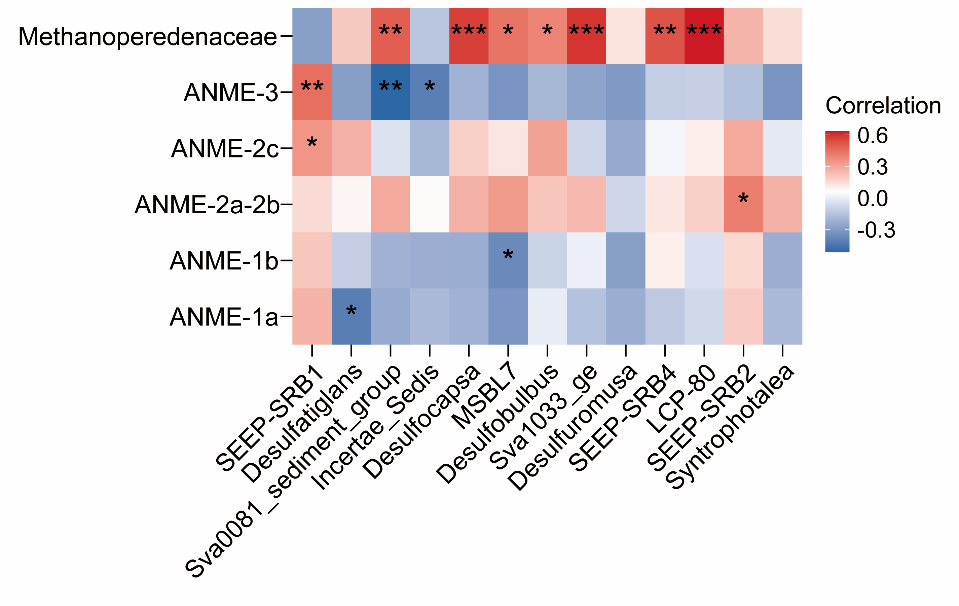


**Fig. S5** Spearman correlation analysis of the relative abundances of ANME and methanogens with SRB in seepage area (ROV5). The asterisks on the circle indicate significant correlations (***, *p* < 0.001; **, *p* < 0.01; *, *p* < 0.05), with red and blue for positive and negative correlations, respectively.


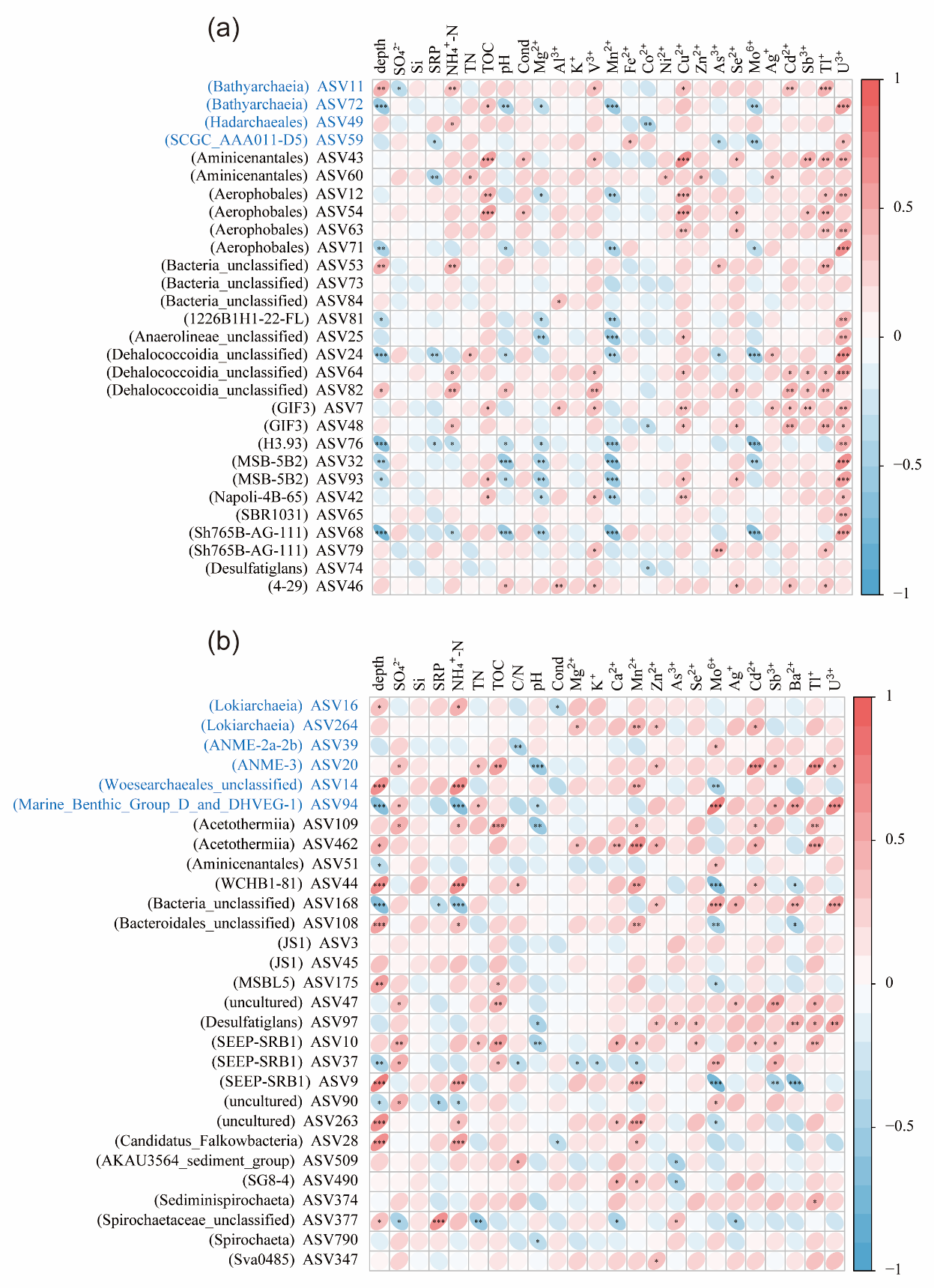


**Fig. S6** Spearman correlation analysis of the most abundant 29 specialized species in non-seepage (ROV3, a) and seepage (ROV5, b) areas with environmental factors. The asterisks on the circle indicate significant correlations (***, *p* < 0.001; **, *p* < 0.01; *, *p* < 0.05), with red and blue for positive and negative correlations, respectively. The archaea (blue) and bacteria (black) are distinguished by color.


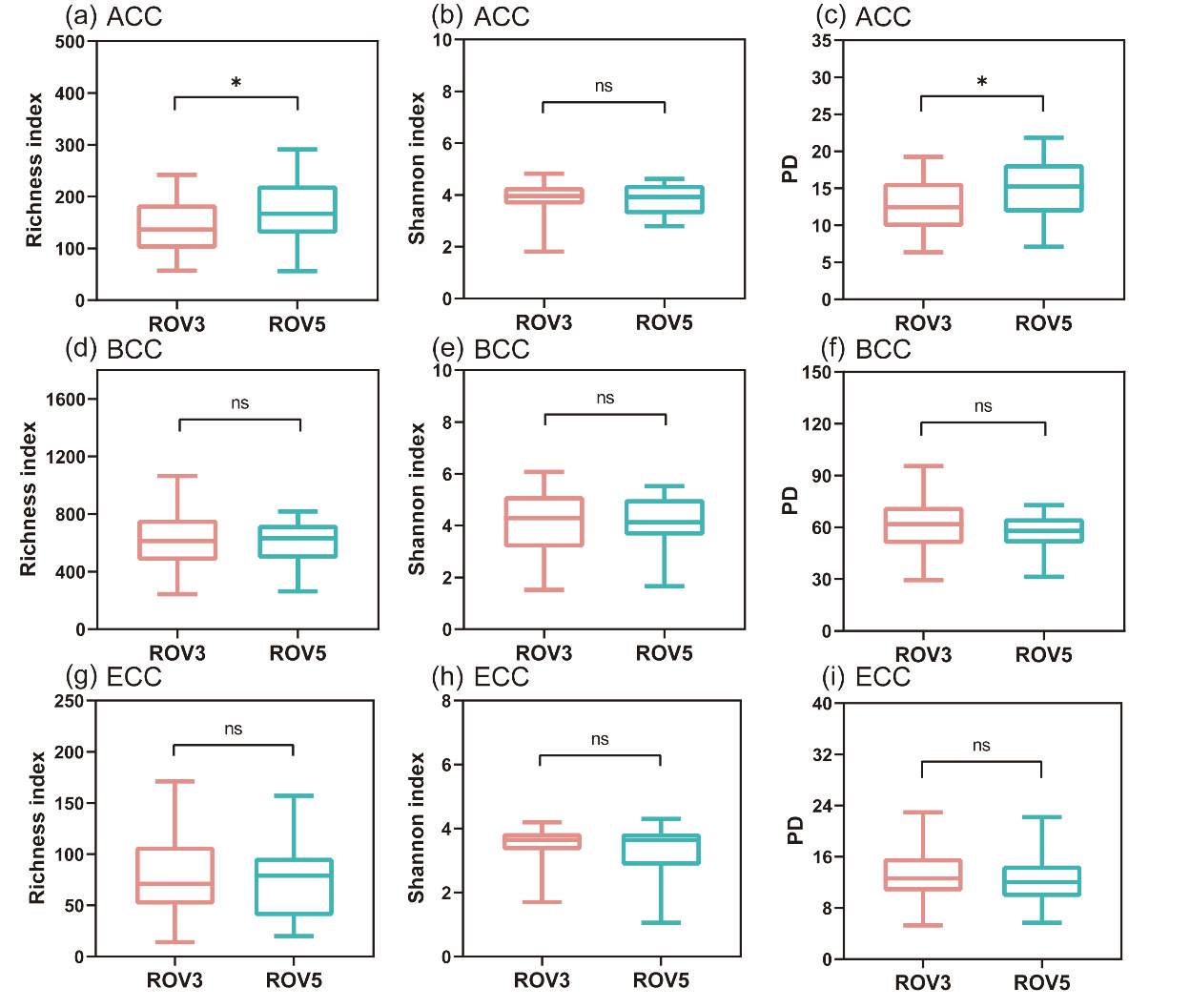


**Fig. S7** Alpha diversity (Shannon, richness, and PD index) of archaeal community composition (ACC), bacterial community composition (BCC), and eukaryotic community composition (ECC) in seepage (ROV5) and non-seepage (ROV3) areas. Symbol indicate significance levels based on *t* tests. *, *p* < 0.05; ns, *p* > 0.05.


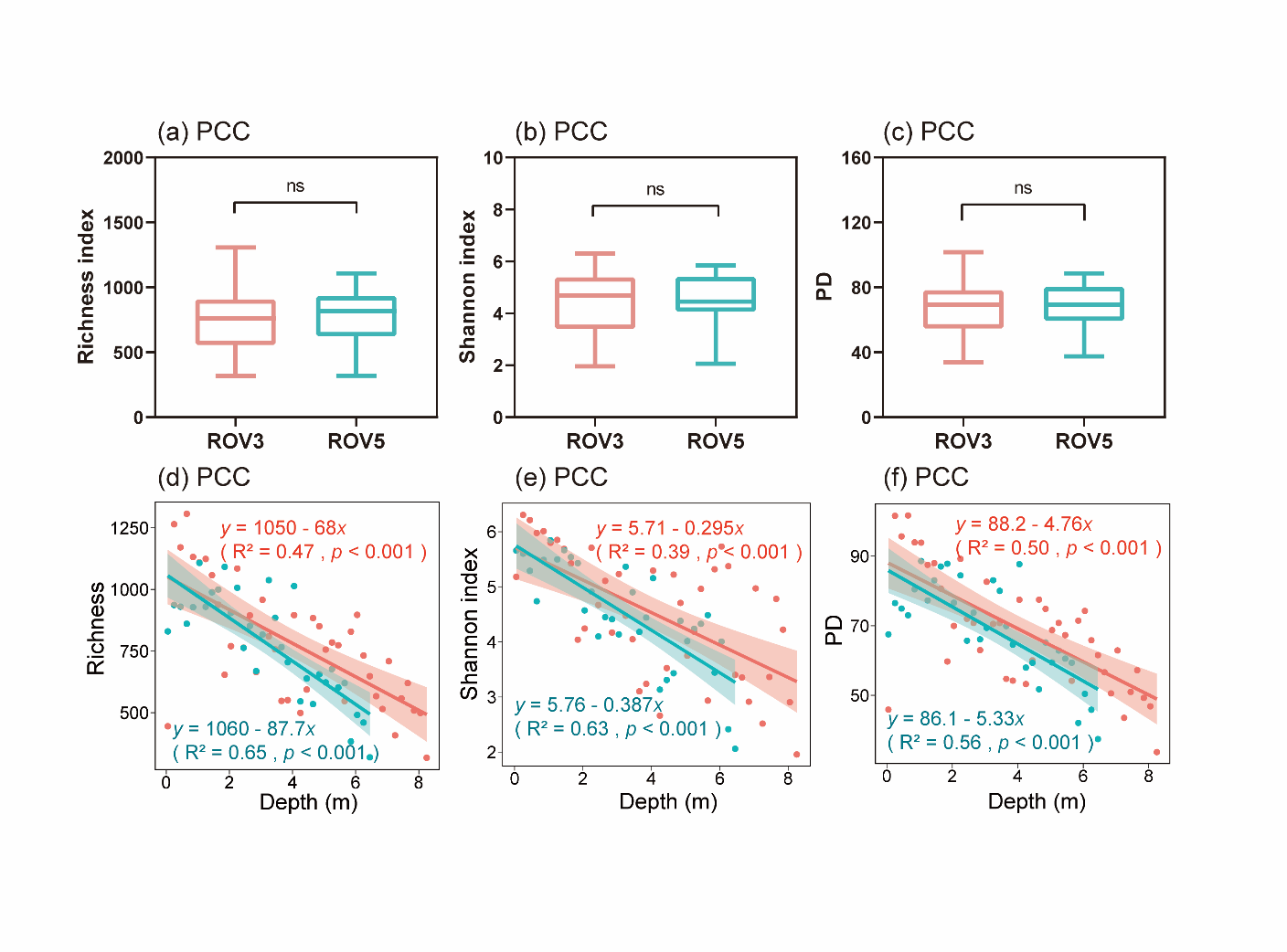


**Fig. S8** Alpha diversity indices (Shannon, richness, and PD index) and their linear correlations with depth for prokaryotic community composition (PCC) in the seepage area (ROV5) and non-seepage area (ROV3). ns, *p* > 0.05.


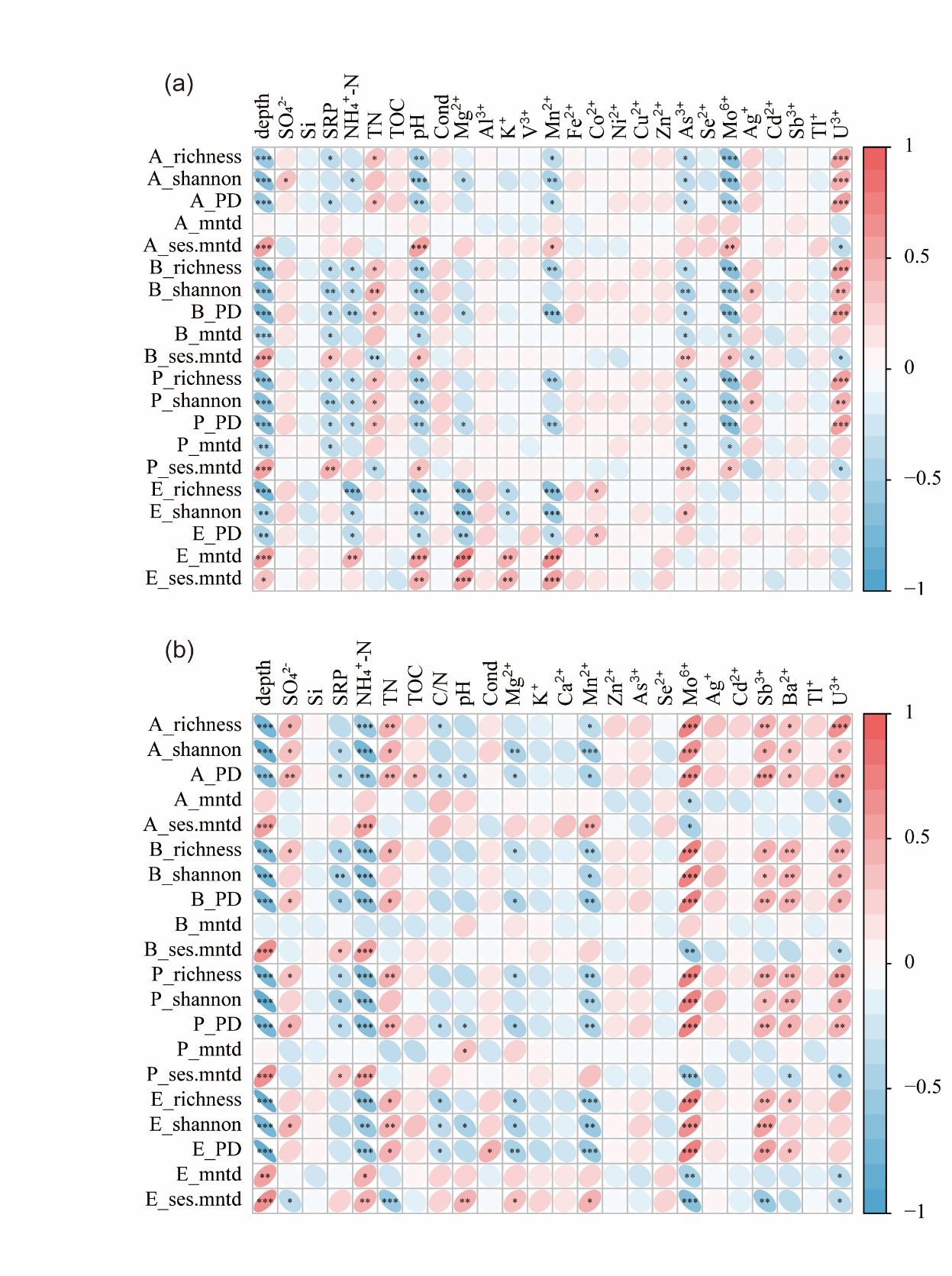


**Fig. S9** Quantification of environmental constraints on richness, Shannon, PD, MNTD, and SES.MNTD in the non-seepage area (ROV3, a) and seepage area (ROV5, b). Asterisks on the circles indicate significant correlations (***, *p* < 0.001; **, *p* < 0.01; *, *p* < 0.05), with red and blue for positive and negative correlations, respectively. The A, B, P and E stand for ACC, BCC, PCC and ECC respectively.


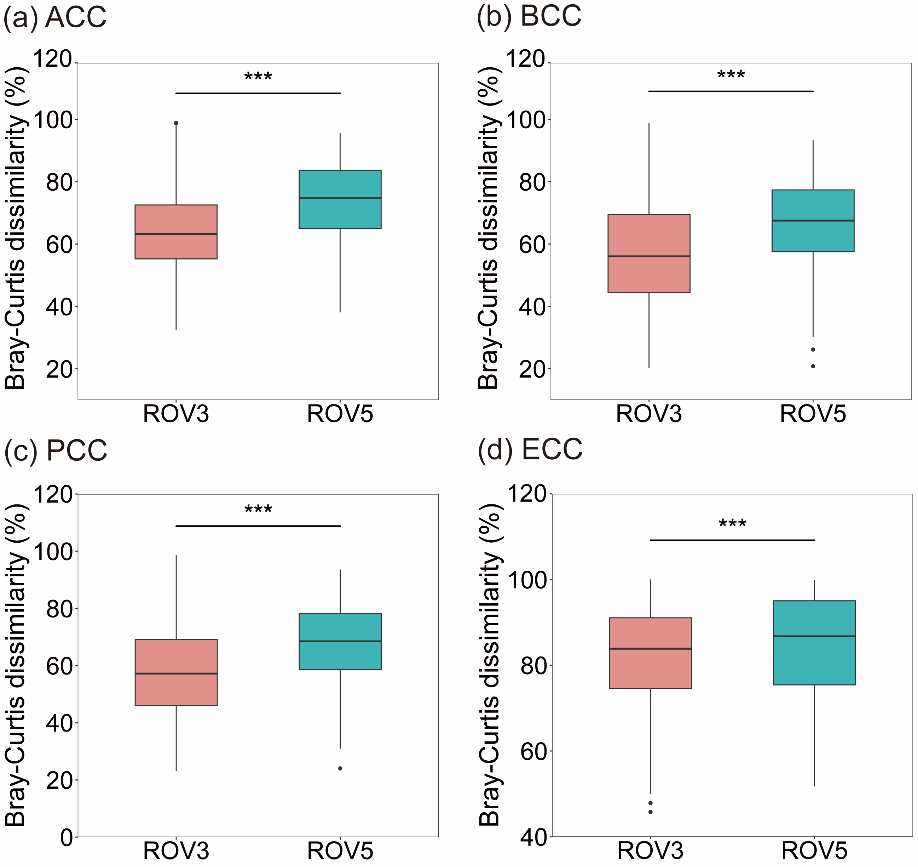


**Fig. S10** Beta diversity of ACC (a), BCC (b), PCC (c), and ECC (d) in the seepage area (ROV5) and non-seepage area (ROV3). Symbols indicate significance levels based on Wilcoxon tests. ***, *p* < 0.001.


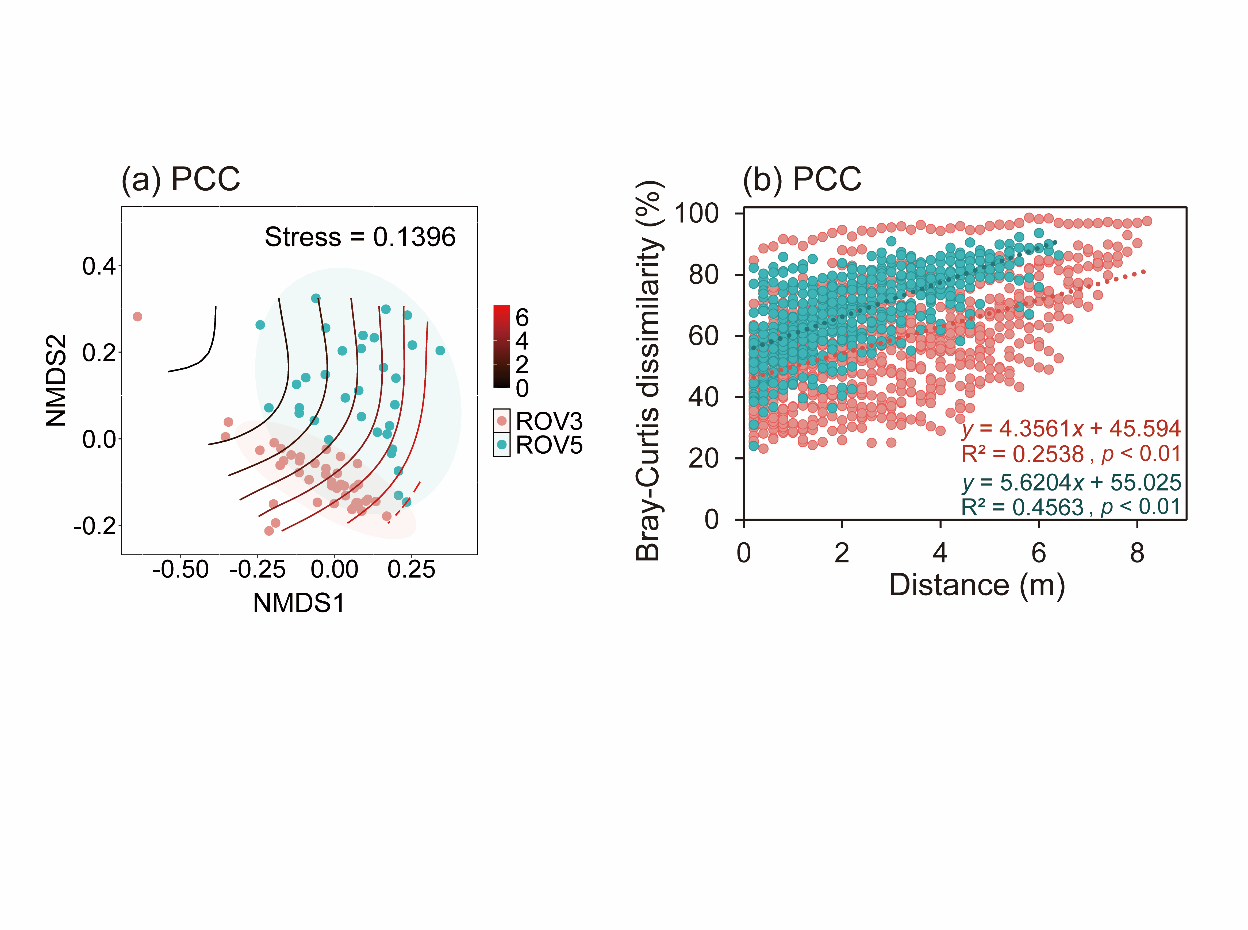


­­**Fig. S****11** Non-metric multidimensional scaling (NMDS) ordinations based on Bray–Curtis dissimilarity of prokaryotic community composition (PCC), along with the relationships between depth distances and PCC dissimilarities in the seepage area (ROV5) and non-seepage area (ROV3).


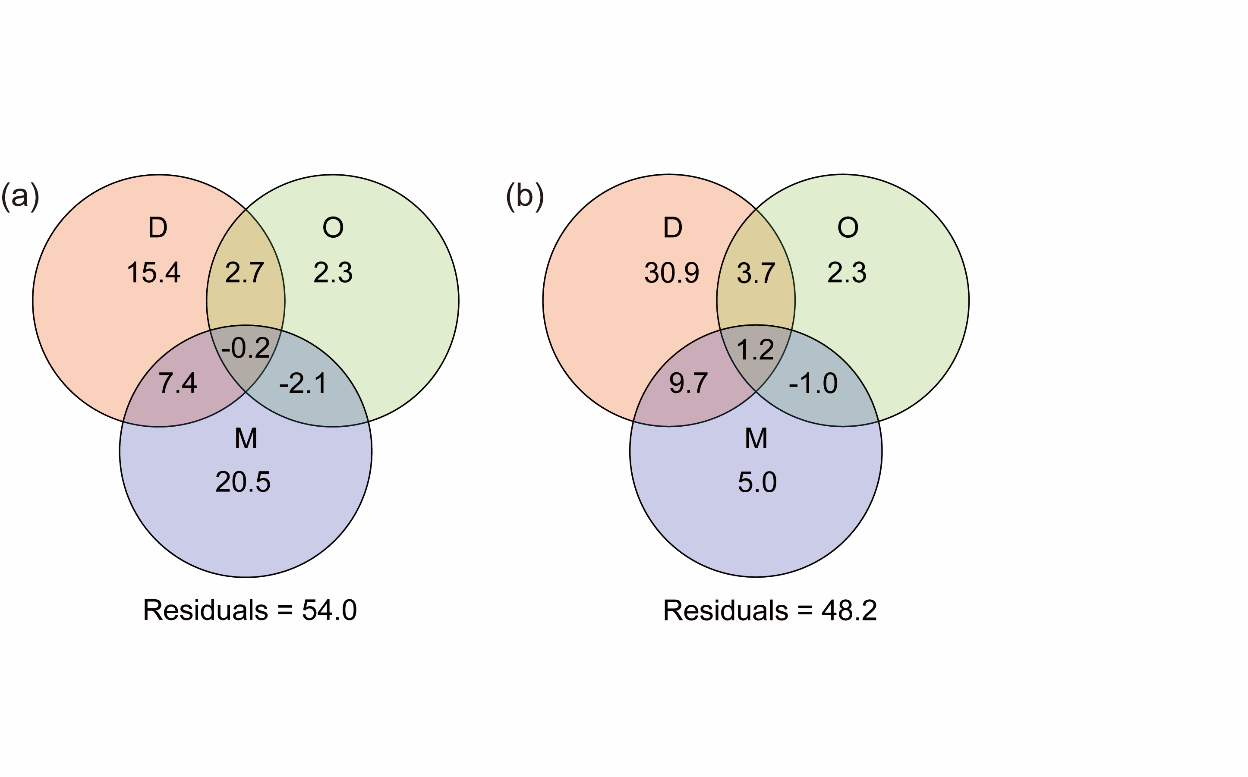


**Fig. S12** Linking environmental parameters to PCC using multiple regression on similarity matrices (MRM) in the non-seepage area (ROV3, a) and seepage area (ROV5, b). The best subsets of environmental parameters for PCC were identified through all-subsets regression: for ROV3, these included depth, SO_4_²⁻, SRP, Mg²⁺, V⁴⁺, Mn²⁺, and Cu²⁺; for ROV5, they included depth, TOC, Mo⁶⁺, Ag⁺, Cd²⁺, Sb³⁺, and U³⁺. D represents depth, O indicates other environmental factors, and M refers to metal environmental factors. Each number reflects the biological variation partitioned into the relative effects of each factor or combination of factors (%).


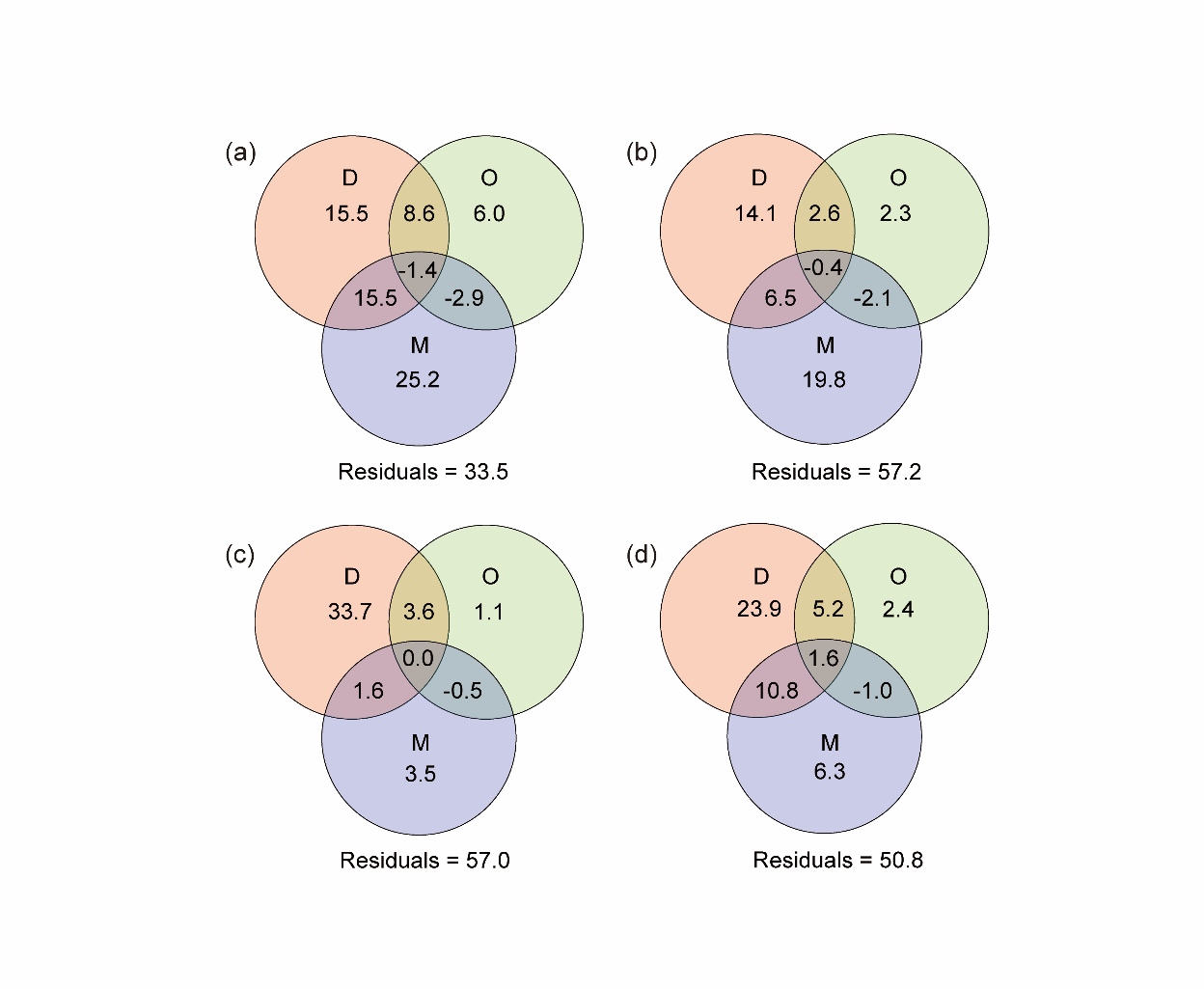


**Fig. S13** Linking environmental parameters to microbial community compositions using multiple regression on similarity matrices (MRM) for ACC (a) and BCC (b) in the non-seepage area (ROV3), and ACC (c) and BCC (d) in the seepage area (ROV5). The best subsets of environmental parameters were identified through all-subsets regression: for ACC in ROV3, these included depth, SO_4_²⁻, SRP, TN, Mn²⁺, Ni²⁺, and Mo⁶⁺; for BCC in ROV3, depth, SO_4_²⁻, SRP, Mg²⁺, V⁴⁺, Mn²⁺, and Cu²⁺ were selected. For ACC in ROV5, depth, SRP, TOC, Mg²⁺, Mn²⁺, Se²⁺, and Ba²⁺ were selected, while for BCC in ROV5, depth, TOC, pH, Zn, Mo⁶⁺, Ag⁺, Sb³⁺, and U³⁺ were identified as key factors. D represents depth, O refers to other environmental factors, and M indicates metal environmental factors. Each number represents the biological variation attributed to the relative effects of individual factors or their combinations (%).


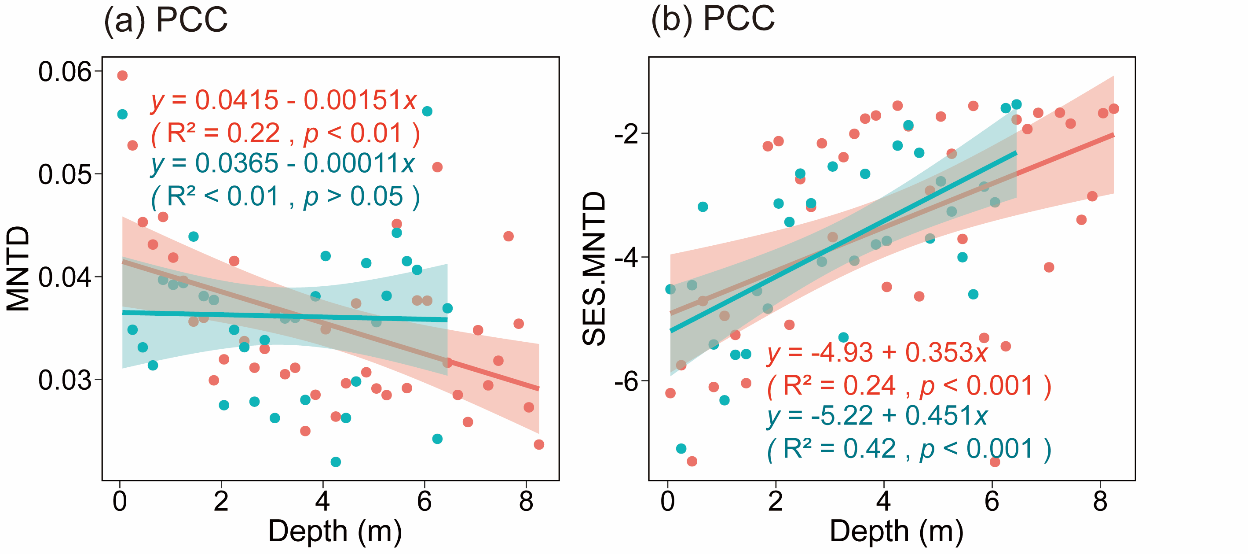


**Fig. S14** Plots of the mean nearest taxon distance (MNTD) vs. depth and the standardized effect sizes of MNTD (SES.MNTD) vs. depth of PCC in non-seepage (red) and seepage (green) areas.


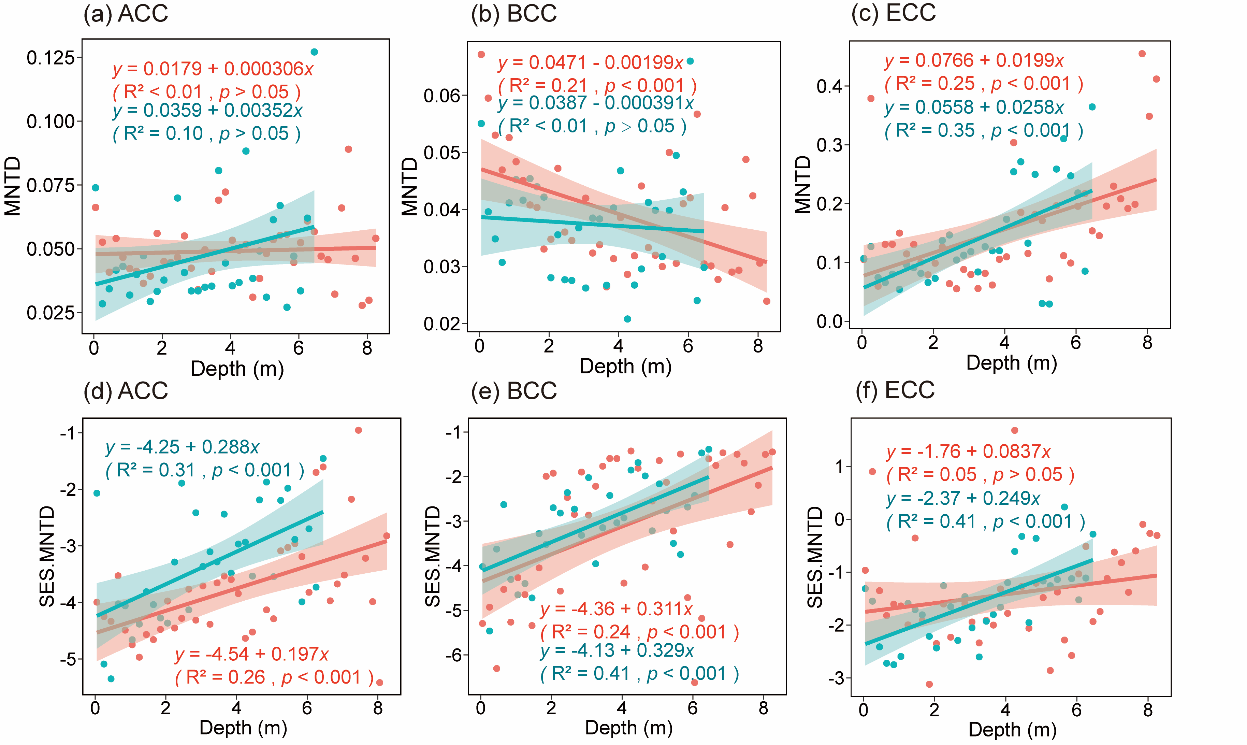


**Fig. S15** Plots of the mean nearest taxon distance (MNTD) vs. depth and the standardized effect sizes of MNTD (SES.MNTD) vs. depth of ACC, BCC and ECC in non-seepage (red) and seepage (green) areas.


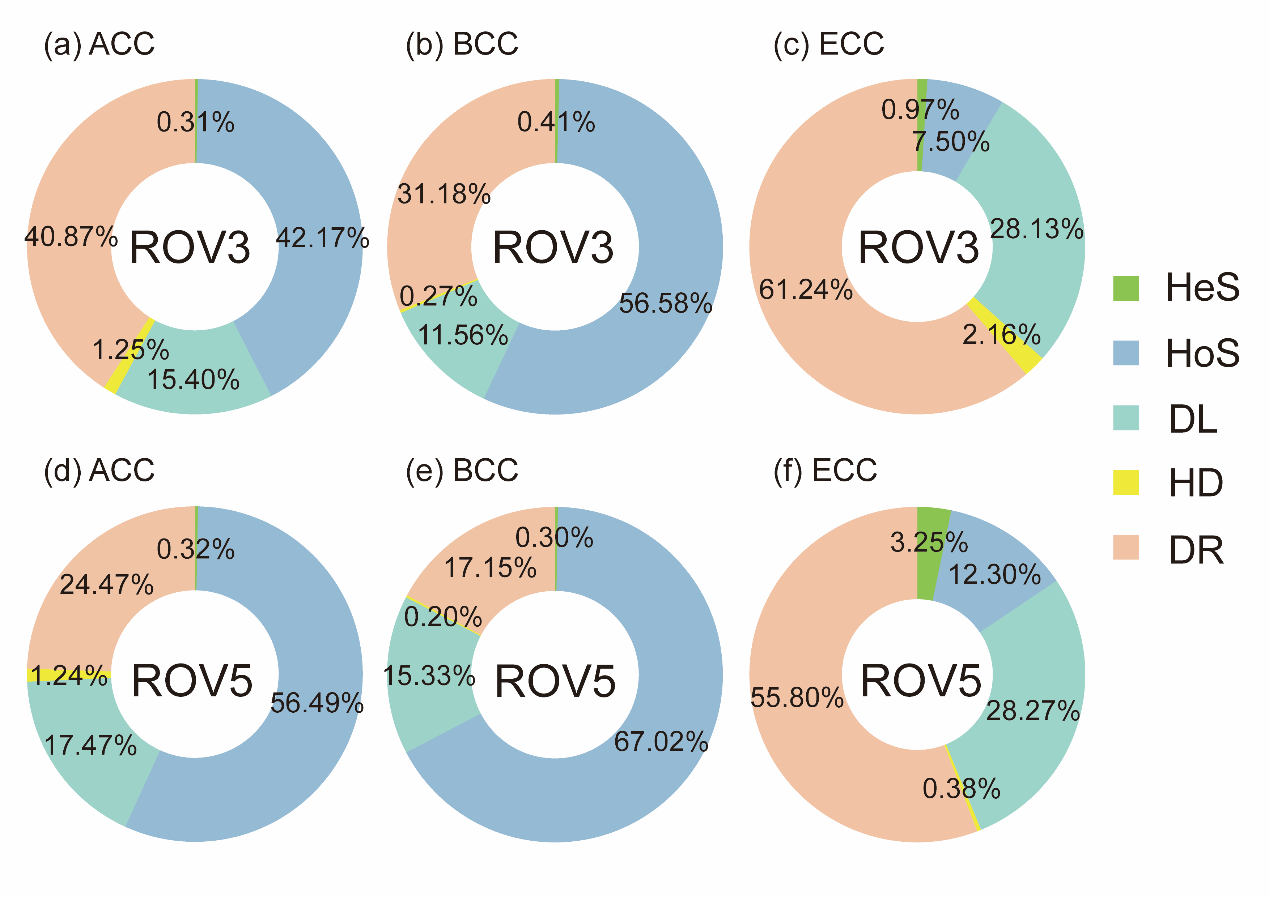


**Fig. S16** The percentage of ACC, BCC and ECC in each assembly process in non-seepage (ROV3) and seepage (ROV5) areas calculated by iCAMP. HeS, heterogeneous selection; HoS, homogeneous selection; DL, dispersal limitation; HD, homogenizing dispersal; DR, drift and others.


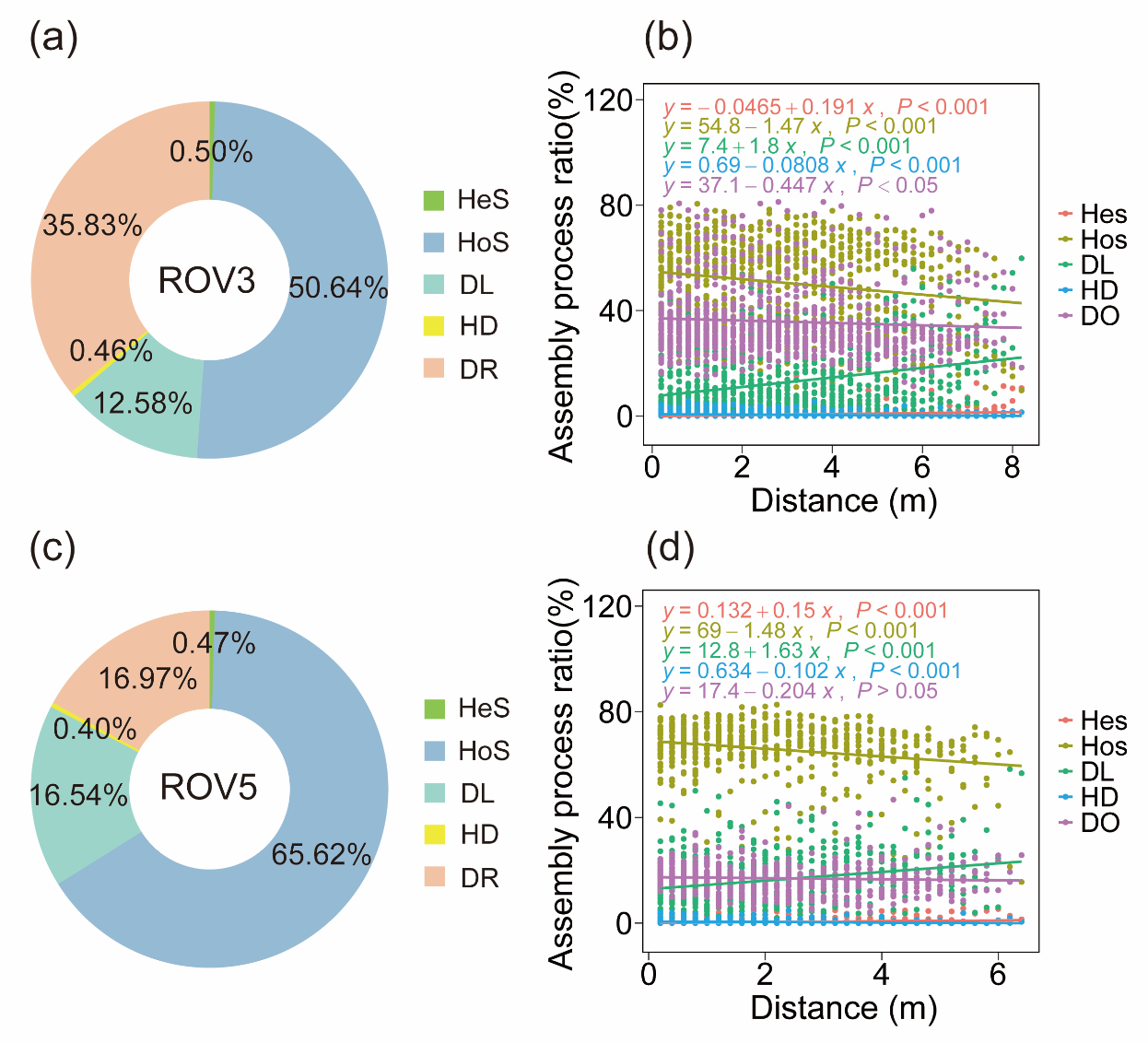


**Fig. S17** The neutral community model (NCM) and their relative percentage of PCC in non-seepage (ROV3) and seepage (ROV5) areas at different assembly processes over geographical distance. HoS, homogeneous selection; DL, dispersal limitation; HD, homogenizing dispersal; DR, drift and others.
